# Thermally treated lanthanum oxide nanoparticles-embedded polyamide composite nanofiber membrane for enhanced mechanical properties and phosphorus adsorption kinetics

**DOI:** 10.3389/fchem.2025.1630889

**Published:** 2025-07-24

**Authors:** Yun Young Choi, David M. Cwiertny, Nosang V. Myung

**Affiliations:** ^1^ Department of Chemical and Biomolecular Engineering, University of Notre Dame, Notre Dame, IN, United States; ^2^ Department of Civil and Environmental Engineering, University of Iowa, Iowa City, IA, United States; ^3^ Department of Chemistry and Biochemistry, University of Notre Dame, Notre Dame, IN, United States

**Keywords:** electrospinning, phosphorous removal, flexible nanofiber, Nylon, lanthanum oxide

## Abstract

Lanthanum oxide (La_2_O_3_) nanoparticles-embedded polyamide 6 nanofiber membranes were electrospun using hexafluoroisopropanol (HFIP) as the solvent. Unlike other solvents such as formic acid and an acetone: trifluoroacetic acid (ace: TFA) mixture, HFIP allowed La_2_O_3_ nanoparticles to remain well-suspended without altering their composition. Various material characterizations confirmed La_2_O_3_ nanoparticles are well embedded in polyamide nanofibers. The phosphorus uptake capacity remains consistent when La_2_O_3_ nanoparticles were embedded in polyamide 6 nanofibers (∼10.4 mg/g) compared to free suspended nanoparticles (∼10.3 mg/g). By optimizing post-thermal treatment, both mechanical strength (e.g., yield strength (σ_y_) from 1.68 × 10^7^ to 2.67 × 10^7^ Pa) and adsorption kinetics (e.g., k_2_ from 2.63 × 10^−2^ to 1.49 × 10^−1^ g/(mg·min)) were improved. This study confirms that post thermal processing can be used to further enhance the mechanical properties of the composite nanofiber membrane while maintaining its phosphate adsorption capabilities and improved adsorption kinetics.

## Highlights

• Lanthanum Oxide (La_2_O_3_) nanoparticles embedded with polyamide 6 nanofibers were electrospun using appropriate solvents.
• Phosphorus update is tuned by adjusting active material content.
• Post thermal treatment enhances the membrane mechanical properties through crystalline tuning while retaining adsorption capacity.
• Optimum mechanical properties (e.g., yield strength and ultimate tensile strength) were achieved by annealing at 120°C for 2 h.

## 1 Introduction

Sustainability is an important concern to support increasing populations through agricultural and industrial advances with minimum environmental impact. Phosphorus is an essential nutrient that has a significant contribution to agricultural development. However, its release through agricultural runoff and industrial waste stream has resulted in eutrophication (Smith et al., 1999), toxic algal blooms (Paerl et al., 2011), oxygen depletion, leading to hypoxic conditions that can suffocate aquatic organism (Ge et al., 2023; Bunce et al., 2018; Durán-Sánchez et al., 2018). Different phosphorus remediation techniques such as enhanced biological phosphorus removal (EBPR) (Izadi et al., 2020), chemical precipitation (Bunce et al., 2018), sorption (Frontiers, 2025), ion exchange (Guida et al., 2021) and membrane filtration (Kim et al., 2008), constructed wetlands (Skinner, 2022) haven been developed to address these issues.

The adsorption method is the most commonly used technique to remediate phosphorus because of its low-cost, high-capacity, and high removal efficiency (Frontiers, 2025). Various adsorbents, such as metal (hyrdro)oxides, metal organic frameworks (MOFs), carbonaceous materials and their derivatives, have been utilized for phosphorus remediation. Most commonly used adsorbents are based on metal (hydro)oxides, including iron (hyrdro)oxides (Ajmal et al., 2018), alumina (Sun et al., 2020), rare earth (hydro)oxides (e.g., cerium and lanthanum oxides) (Kajjumba and Marti, 2022; He et al., 2022). More specifically, lanthanum hydro (oxides) have a high theoretical phosphorus capacity (520.79 mg P/La^+3^) due to strong rare earth element-phosphate bonds (REE-PO_4_) with wide pH stability (3.0–10.0), low toxicity, low environmental impact, and high chemical stability. Because of these advantages, REE hydro (oxides) have been incorporated to various functional carrier matrix (e.g., biochar (Luo et al., 2023) and silica (Othman et al., 2019)) to improve adsorption capacity. To further enhance the adsorption kinetics and capacity, researchers have been engineering the REE to reduce their dimensions to promote higher surface adsorption sites. For examples, Qiu et al. immobilized nanosized lanthanum (hydr)oxides within quaternary-aminated wheat straw that increased the specific surface area by 24 times from 3.27 m^2^/g to 78.76 m^2^/g and showing about 3.4 times improvement in P uptake (Qiu et al., 2017). Fang et al. synthesized lanthanum hydroxide nanorods with different aspect ratio and showcased the high phosphorus update (∼160 mg P/g to 170 mg P/g) (Fang et al., 2018). Although reducing the adsorbent size enhances the removal rate and capacity, utilizing nanosized adsorbents necessitate additional separation/removal steps. Furthermore, the issue of nanosized materials loss must be addressed (Nalbandian et al., 2022).

Nanoparticle embedded polymer nanofiber-based membranes overcome the limitation of suspended nanoparticles to eliminate the need of additional separation/removal steps. Additionally, chemically functionalized composite nanofiber membranes exhibit distinct features of dual functionality, serving as both physical and chemical filtration methods (Rajak et al., 2019; Rajak et al., 2022). For example, while the three-dimensional porous membrane provides physical separation, active adsorbents can be embedded into the carrier matrix to remediate various pollutants (Nalbandian et al., 2022). Electrospinning is unique one-pot synthesis techniques to fabricate composite nanofiber membranes, allowing for controlled dimension and morphology by adjusting electrospinning solution compositions and electrospinning parameters (Wang et al., 2021; Greenstein et al., 2019; Peter et al., 2017; Choi et al., 2024; Ding et al., 2022).

Polyamide, also known as Nylon, has been extensively utilized as a water filter membrane because of its low-cost, high chemical resistance, and excellent mechanical durability which enhances its lifetime in various water treatments (Guibo et al., 2013; De Schoenmaker et al., 2013; Roslan et al., 2018). Its hydrophobic nature minimizes biofouling (Aziz et al., 2024). Polyamide can be classified into aliphatic polyamides, polyphthalamides, and aromatic polyamides, according to the main chain composition (Rayjadhav et al., 2024). Polyamide 6 (PA6), one of the most common types of polyamides, has exceptional mechanical strength, great flexibility, and high chemical resistance (Su et al., 2007). PA6 has two common crystalline phases, α and γ phases, that can coexist depending on the processing conditions that impact its mechanical and physical properties (Liu et al., 2007; Ahmad et al., 2021; Zhang et al., 2011).

In this work, mechanically durable lanthanum oxide embedded PA6 composite nanofibers were electrospun and applied toward phosphorus remediation. To synthesize composite nanofibers in a single step, various solvents were examined. Furthermore, membrane strength were further improved through fine-tuning of crystallinity and crystal phase during the post thermal process. During thermal treatment, the crystal structure of electrospun PA6 nanofiber altered from γ crystal phase to thermodynamically stable α phase, resulting in significant enhanced of mechanical properties such as yield strength and ultimate tensile strength. Additionally, post thermally treated composite nanofiber membrane retained adsorption capacity and improved adsorption kinetic compared to as-spun membrane.

## 2 Materials and methods

### 2.1 Materials

Polyamide6 (PA6) and tetra-n-butylammonium bromide (TBAB; ≥98%) were obtained from Sigma-Aldrich whereas trifluoroacetic acid (TFA), acetone, potassium dihydrogen phosphate (KH_2_PO_4_; 99.3%), sulfuric acid, formic acid, and ascorbic acid (99.4%) were purchased from Thermo Fisher Scientific. Lanthanum oxide (La_2_O_3_, >99.99% 30–50 nm) nanoparticles were purchased from ACS materials. 1,1,1,3,3,3-Hexafluoro-2-propanol (HFIP) was purchased from Oakwood Chemical. All materials were used without further treatment.

### 2.2 Preparation of electrospinning solutions

The most common solvents to dissolve PA6 include formic acid, mixture of acetone and strong acid such as trifluoracetic acid (TFA), and 1,1,1,3,3,3,3-Hexafluoro-2-propanol (HFIP) (Heikkilä and Harlin, 2008). The solution was then sonicated to disperse the La_2_O_3_ homogenously. PA6 was then added and stirred until PA6 pellets were fully dissolved. Lastly, TBAB was added to the solution and stirred overnight for homogeneous solution for electrospinning.

### 2.3 Solution characterization

The solutions properties (viscosity, surface tension and electrical conductivity) of the solution were measured. The viscosity was measured using a CPA-40 spindle connected to a Brookfield DV2THB viscometer where the solution viscosity was determined to be independent of the shear rate. Thus, the viscosity was measured at 95% torque at each rotational speed ranging from 0.5 rpm to 200 rpm. An automatic surface tensiometer (Shanghai Fangrui Instrument, QBZY-1) with platinum-coated plate was used to measure the surface tension. Solution electrical conductivity was measured using an electrical conductivity probe from Apera Instruments (Al1311, K = 0.1) connected to EZO conductivity circuit (Atlas Scientific), on Tentacle T3 using Raspberry Pi (Whitebox T3, Mkll). All solution property measurements were taken at room temperature prior to electrospinning to correlate them closely to the resulting nanofiber properties.

### 2.4 Electrospinning

Electrospinning was carried out by injecting the prepared solution through a 5-mL BD Luer-Lok syringe with a 25-gauge stainless steel needle using a syringe pump (New Era, NE-100). High-voltage power supply was used to apply the voltage between the needle tip and drum collector. The drum collector was wrapped with aluminum foil and rotated around 400 rpm. Electrospinning and environmental conditions including applied voltage, feed rate, temperature, and absolute humidity were fixed at 12 kV, 0.25 mL/h, 23 ± 1°C, and 0.008 ± 0.001 kg H_2_O/kg dry air, respectively. To investigate the effect of post thermal treatment, the as-spun nanofiber samples were annealed in a tube furnace (Thermo Scientific Lindberg/Blue M Mini-Mite TF55030) at varying temperatures (i.e., 80, 100, 120, 140, and 200^°^C) for 2 h in air.

### 2.5 Nanofiber characterizations

Morphology of the as-spun nanofiber was observed with a scanning electron microscope (Prisma E SEM, Thermo Fisher Scientific, United States). Prior to analysis, a thin layer of gold was sputtered using Electron Microscopy Sciences 575X over the samples at 20 mA for 30 s to minimize surface charging. Obtained SEM images were imported to ImageJ software to measure the average fiber diameter, which was obtained by measuring the diameter of 50 unique nanofibers. The bead density was also calculated by dividing the total number of beads from a single SEM image by the total area of the image. Fiber fraction was determined by the proportion of nanofibers in the total product, which could include beads and clumps. Transmission electron microscopy (TEM) samples were collected by placing a carbon-coated copper grid directly in front of the drum collector for 1 min during electrospinning. TEM images were captured using 300 (S)TEM Ceta™.

The crystal structures were characterized by using a powder X-Ray diffractometer (Rigaku Miniflex 6 g). Multiple 2 × 2 cm^2^ sheets were coherently layered to ensure X-ray detection of the sample. Data collection was performed at room temperature at *λ* = 1.5406 Å over a 2θ range of 10°–80° with a step size of 0.025°. The molecular structures were observed with Perkin Elmer Spotlight 200 Fourier-transform infrared (FT-IR) spectrometer. For FT-IR, approximately 0.5 × 0.5 cm^2^ of sample was cut and placed in a pass-through sample holder. FT-IR spectra were then obtained by scanning from 450 to 4,500 cm^−1^ at a resolution and scan increment of 1 cm^−1^.

Differential scanning calorimetry (DSC) was performed by using a DSC 2500, TA Instruments, United States. The accurately weighed samples were placed into Al crucible, and which was used for the analysis. The samples were first heated to 250°C, at a rate of 10^°^C/min. The sample was then cooled down to 0°C, at a rate of 10 °C/min and reheated to 250°C, at a rate the same rate. This cooling and reheating cycle was repeated twice. Mechanical properties were examined using a Discovery hybrid rheometer (DHR-30, TA Instruments, United States) attached with RH Linear Tension Rectangular Fixture. Composite nanofiber samples were placed between the plates and the samples were pulled apart at a constant linear rate of 1.0 mm per second until 50 mm is reached at room temperature. Mechanical properties were determined using software provided by the manufacturer (TRIOS, TA Instruments).

### 2.6 Phosphate adsorption studies

Batch adsorption experiments were undertaken to determine the adsorption capacity of phosphate. Various initial P concentrations ranging from 2 to 20 mg/L were tested with adsorbent dose of 1 g/L of La_2_O_3,_ relative to the solution volume, at room temperature. The solution was collected using a 3-mL BD Luer-Lok syringe with a syringe attached (0.22 μm, PTFE Teflon filter) at different time intervals (0.5, 1, 2, 5, 10, 15, 240, and 1,440 min). The phosphate concentration of collected samples was measured at 880 nm with a UV-Vis spectrophotometer (Agilent Cary 60) based on the ascorbic acid molybdate blue method (Wang et al., 2021).

The equilibrium adsorption capacity (q_e_) was calculated using Equation 1:

$$q_{e}=\frac{\left(C_{o}-C_{e}\right)\cdot V}{m}$$ (1)

where q_e_ (mg/g) is the adsorption capacity at time t, C_0_ and C_e_ are the initial and equilibrium phosphate concentrations in mg/L, respectively. V is the volume of the solution in L, and *m* is the mass of the composite nanofiber mat in g.

The adsorption kinetic was investigated using the pseudo first order and pseudo second order equations (Equations 2, 3, respectively).

$$q_{t}=q_{e}\cdot \left(1-\exp\left(k_{1}\cdot t\right)\right)$$ (2)

$$\frac{t}{q_{t}}=\frac{1}{\left(k_{2}q_{e}^{2}\right)+\frac{t}{q_{e}}}$$ (3)

where the rate constant of pseudo first order adsorption as k_1_ (min^−1^) and k_2_ as rate constant of pseudo second order adsorption (g·mg^−1^ min^−1^); q_e_ is the amount of phosphate adsorption at equilibrium (mg/g); q_t_ is the amount of phosphate adsorption at time t (min) in mg/g. Equations 4, 5 were used to determine the adsorption isotherms using Langmuir and Freundlich isotherm models:

$$q_{e}=\frac{\left(q_{\max}K_{L}C_{e}\right)}{\left(1+K_{L}C_{e}\right)}$$ (4)

$$q_{e}=K_{F}C_{e}^{1/n}$$ (5)

where q_max_ is the maximum adsorption capacity (mg/g); C_e_ is the equilibrium phosphate concentration (mg/L); *n* is the parameter of the Freundlich adsorption isotherm; and K_L_ (L/mg) and K_F_ ((mg/g) · (L/mg)^1/n^) are the equilibrium constants related to the Langmuir and Freundlich adsorption isotherms, respectively.

## 3 Results and discussion

### 3.1 Effect of solvents on solution properties

**FIGURE 1:**
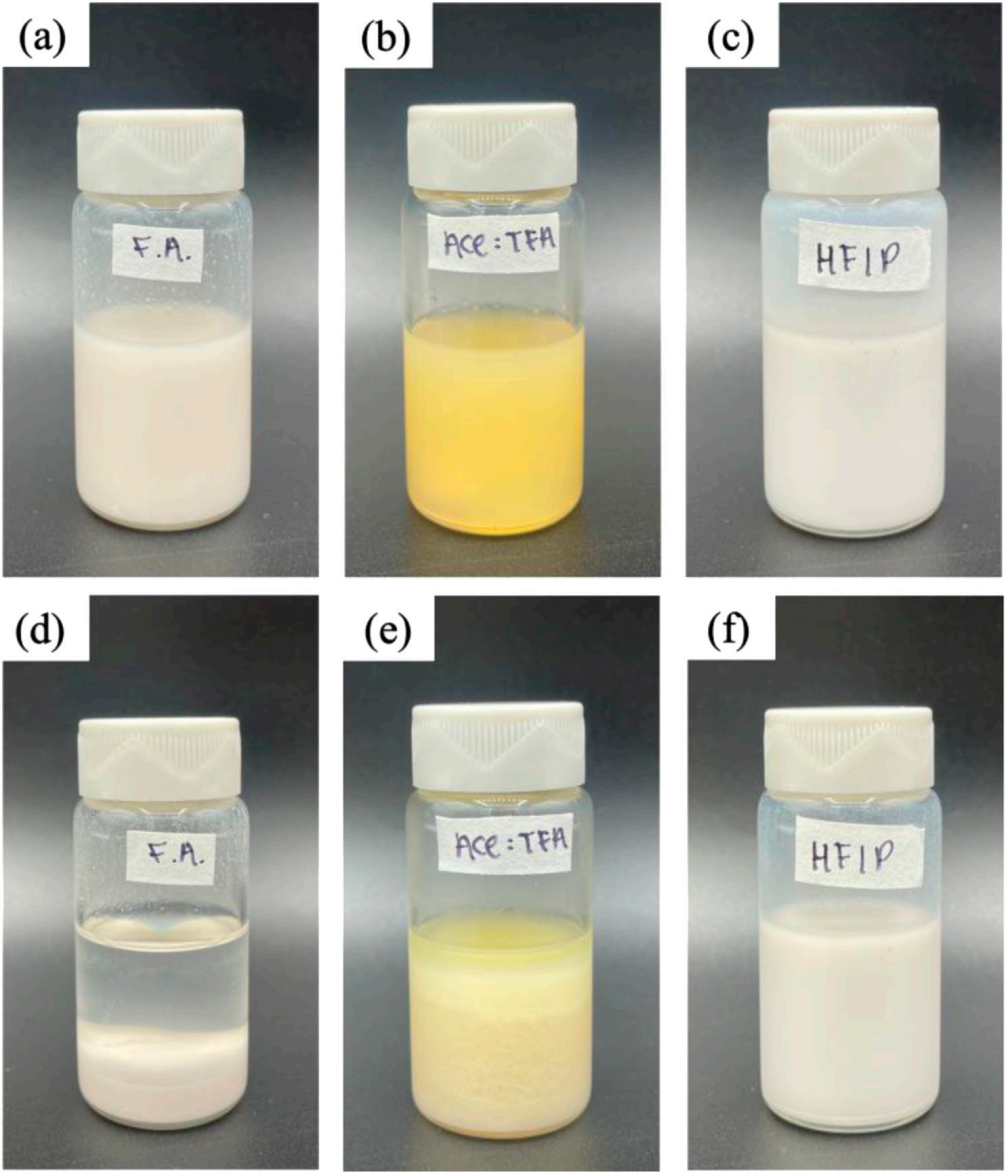
Images of one-pot electrospinning solutions for PA6/La_2_O_3_/TBAB composite with various solvents: formic acid (F.A.), Ace: TFA (60:40 mol%), and HFIP at time 0 are shown in **(a–c)**, respectively. The solutions after 5 h are shown in **(d–f)**.

Initially, La_2_O_3_ nanoparticles in the Act:TFA mixture showed homogeneity (Figure 1a). However, La_2_O_3_ nanoparticle settled within a few hours (Figure 1d). Since active adsorbents settled in the electrospinning solution, a limited amount of La_2_O_3_ nanoparticles may have embedded into electrospun nanofibers. To ensure that the synthesized membrane contains all the ingredients added to the solution, the solvent must be altered. As mentioned previously, there are other solvents that are known to be compatible with PA6: e.g., formic acid and HFIP. Thus, these solvents were also used as electrospinning solutions. Figures 1b,c shows optical images of as-prepared solutions after the solution was stirred overnight which appeared to be homogeneous. Figures 1d—f shows the images after being left to rest state for 5 h. As shown in Figures 1b,d, the color of formic acid based solutions was yellow compared to milky white for Act: TFA and HFIP based solutions. This might be attributed to the interacting between La_2_O_3_ nanoparticles and formic acid. The solution made with HFIP remained homogeneous after 5 h (Figure 1f).

To understand the solvent effects, all three solutions are used to electrospun. The mass of PA6, lanthanum oxide nanoparticles and TBAB kept constant while the solvents were varied to understand the solvent effect. As expected, solution properties (i.e., viscosity, surface tension and electrical conductivity) varied significantly depending on the solvent used (Supplementary Table S1).

**FIGURE 2:**
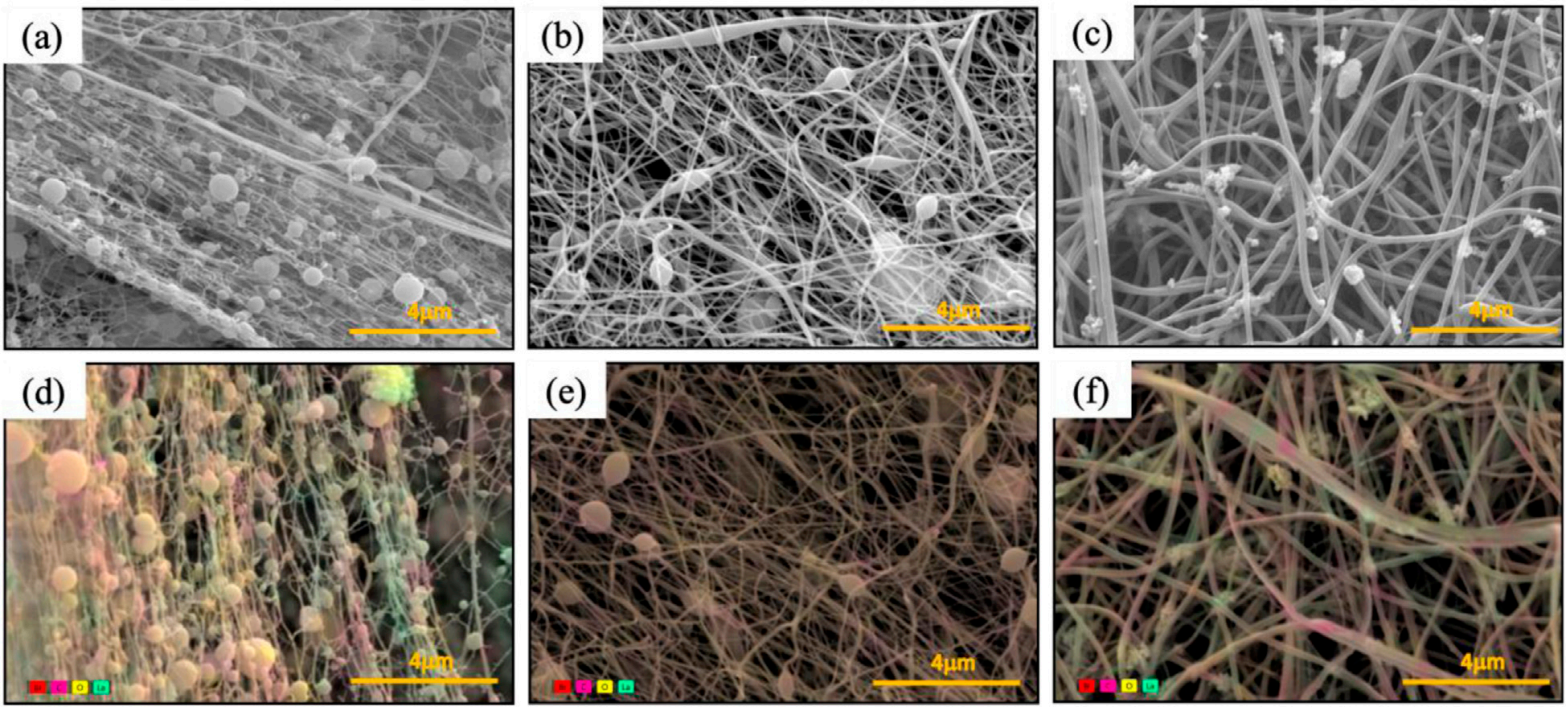
SEM images **(a–c)** and color SEM **(d–f)** of PA6/La_2_O_3_/TBAB NF membrane with various solvents: **(a,d)** formic acid, **(b,e)** Ace: TFA (60:40 mol%), and **(c,f)** HFIP. Presence of lanthanum (shown in teal) and bromide (shown in red) against carbon of polyvinyl pyrrole (shown in pink).

There are various factors to consider when electrospinning nanofibers. First, is the solution electrospinnable with a high fiber fraction instead of electrospraying. Second, all the components that are in the solution should be electrospun onto the nanofiber membrane except the solvents. As shown in Figure 2, all three solvents resulted in electrospinning over electrospraying with a high fiber fraction. At first glance, there are fiber diameter and bead density variations depending on the solvent used. The formic acid-based solution has the highest viscosity (i.e., 1,664 cP) but resulted in the smallest average fiber diameter of 65 nm (Figure 2a). Previous studies have reported that a high viscosity results in a higher average fiber diameter, but the opposite trend was shown for nanofibers synthesized from formic acid. Additionally, higher bead density (0.16 beads/μm^2^) was observed compared to when other solvents were used. The mixture of acetone and Act:TFA based solution has a viscosity of 90.1 cP and resulted in nanofiber mats with an average fiber diameter of 97 nm and a fiber fraction of 0.93 μm^2^/μm^2^ (Figure 2b). The HFIP based solution has a viscosity of 69.2 cP and resulted in nanofiber mats with an average fiber diameter of 162 nm and a high fiber fraction of 0.98 μm^2^/μm^2^ (Figure 2c). In addition to average fiber diameter and fiber fraction, the diameter distribution is shown in Supplementary Figure S1.

To determine the distribution of La_2_O_3_ nanoparticles within nanofibers, energy dispersive areal mapping was overlaid on top of the secondary electron scanning electron microscope image (Figures 2d–f). The presence of lanthanum was shown in teal and bromide is shown in red against the carbon of PA6 shown in pink. Compared to the nanofiber obtained from the HFIP based solution, the areal mapping of the nanofiber obtained from Act:TFA based solution shows lower intensity of lanthanum (Figure 2e) which was expected due to agglomeration and settling of La_2_O_3_ nanoparticles. Unlike other solvents, composite nanofibers synthesized from the HFIP solvent show well distributed La_2_O_3_ nanoparticles embedded in PA6 matrix.

**FIGURE 3:**
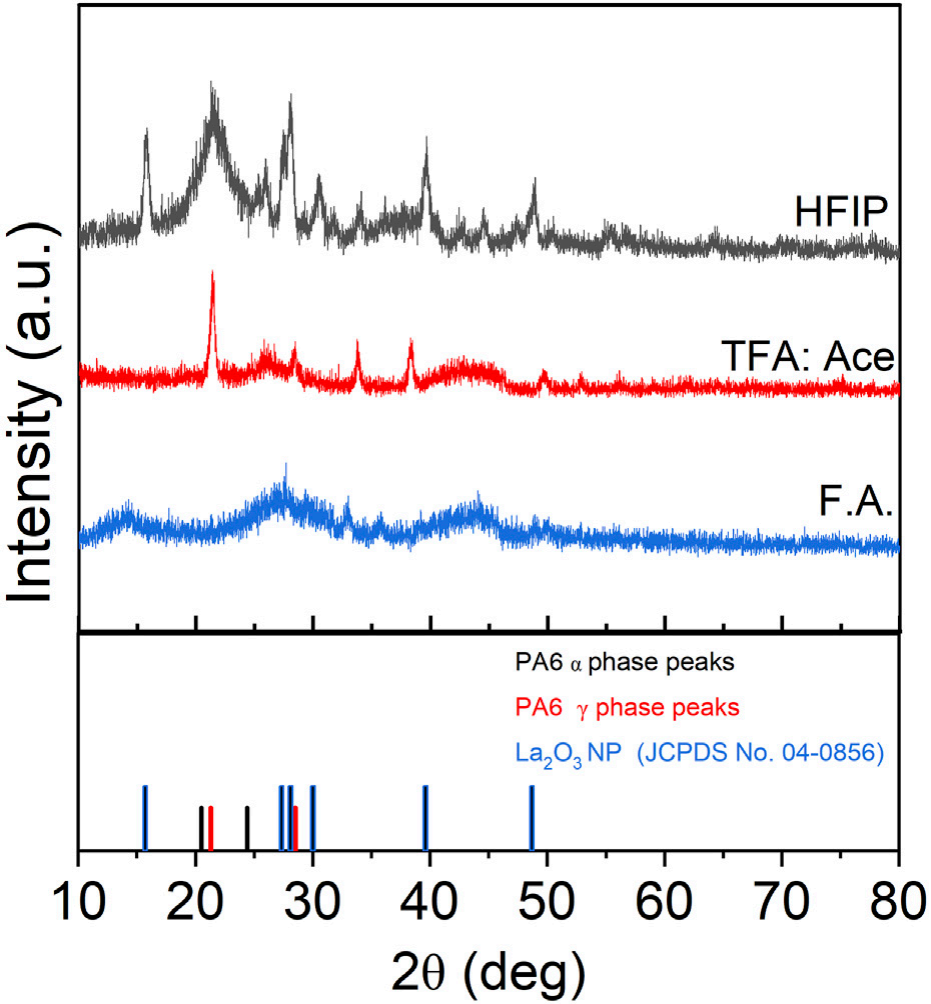
XRD of PA6/La_2_O_3_/TBAB NF with various solvents.

The stable dispersion observed for La_2_O_3_ in HFIP compared to other solvents might be attributed to the fact that La_2_O_3_ reacts with formic acid and TFA, but not in HFIP. Specifically, La_2_O_3_ reacts with formic acid to form lanthanum formate and with TFA to form lanthanum triflate. These reactions prevent La_2_O_3_ from remaining in its original form prior to the addition of other chemicals in the tri-composite solution. In contrast, the absence of such a reaction in HFIP likely preserves the oxide form, resulting in a more stable dispersion. This is supported by the XRD pattern shown in Figure 3, which compares composite nanofibers synthesized using different solvents. As shown in the figure, the XRD patterns differed significantly based on the solvent. The composite nanofiber synthesized from HFIP showed dominant peaks at 15.7, 27.4, 28.1, 30.0, 39.6, and 48.7° which are attributed to the standard XRD pattern of lanthanum oxide (JCPDS 04—0856). The sample that was synthesized using the Act:TFA mixture solvent showed a few diffraction peaks (28.1° and 48.7°) that matched to La_2_O_3_, but the diffraction pattern also showed other peaks that could not be correlated to La_2_O_3_ nanoparticles. The composite nanofibers electrospun from formic acid did not show any diffraction peaks indicating that the La_2_O_3_ nanoparticles were not presented.

### 3.2 High resolution transmission electron microscope (HR-TEM) with elemental mapping

**FIGURE 4:**
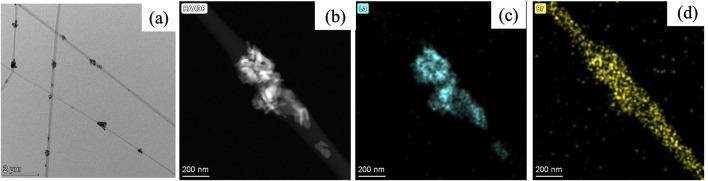
TEM image **(a)** and mappings of PA6/La_2_O_3_/TBAB composite NF showing **(b)** HAADF image, **(c)** lanthanum, and **(d)** bromide. The elemental mapping indicates that bromide from TBAB overlaps with lanthanum, but also demonstrates a homogeneous dispersion across the NF, without surface segregation of lanthanum particles.

Figure 4a shows the HR-TEM image of representative PA6/La_2_O_3_/TBAB composite nanofiber (PLT NF) capturing clusters of lanthanum oxide nanoparticles within PA6 nanofibers. In addition to HR-TEM, elemental mappings of nanofibers were conducted to further understand the distribution of La_2_O_3_ nanoparticles and TBAB within the composite nanofibers. Elemental mapping of lanthanum (Figure 4c) and bromide (Figure 4d) was obtained from the high-angle annular dark field (HAADF) image shown in Figure 4b. La mapping shows that the La_2_O_3_ are well embedded within PA6 nanofiber (Figure 4c). Bromide mapping from TBAB shows overlapping with La but also distributed across the NF. Previous work showed that TBAB was used to disperse the aggregated nanoparticles as well as to help smaller Fe_2_O_3_ nanoparticles enrich themselves to the surface of nanofibers. However, aggregated La_2_O_3_ nanoparticles can be seen within nanofibers in Figure 4a suggest that TBAB did not act as a surfactant or help with surface enrichment of adsorbents. This may be attributed to the fact that the La_2_O_3_ nanoparticles (30—50 nm) are much larger compared to previously reported work (i.e., Fe_2_O_3_ nanoparticles with an average particle size of 3 nm).

### 3.3 Phosphate removal batch testing

**FIGURE 5:**
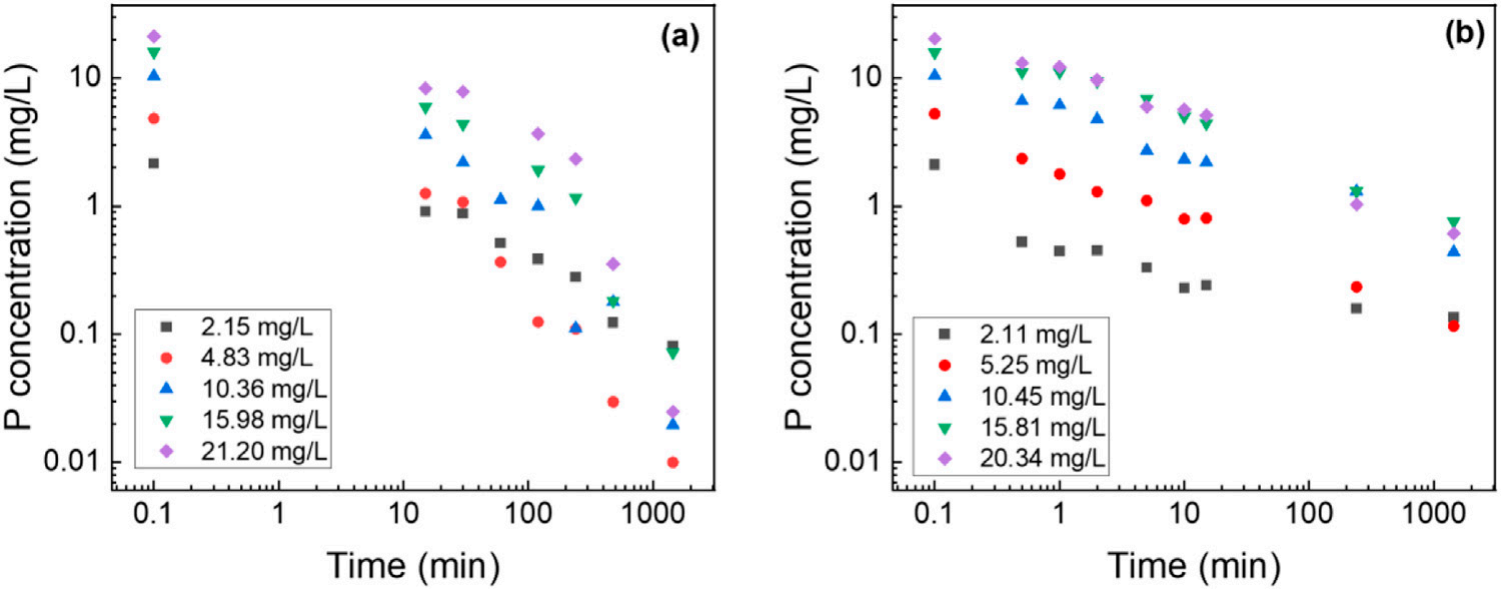
Log-log graph of P adsorption concentration of lanthanum oxide **(a)** NP and **(b)** composite NF. Adsorbent dosage: 1 g/L.

To benchmark the adsorption capacity and kinetics of composite nanofiber membranes, suspended metal oxide nanoparticles were tested as a control to determine the maximum adsorption capacity which showed the P adsorption capacity of 21.6 mg/g (Supplementary Figure S2). The results from this preliminary testing served as the control for evaluating the PA6/La_2_O_3_/TBAB composite nanofibers synthesized using various solvents. Figure 5 shows the batch P removal testing of the composite nanofiber. As shown in the figure, the composite nanofiber membrane showed 97.1% phosphate removal compared to suspended nanoparticles which showed 98.3% removal with an initial concentration of 20.3 mg/L. Our previous work investigated the effect of metal oxide content variation—specifically with iron oxide as the active ingredient—on phosphorus removal efficiency (Wang et al., 2021; Choi et al., 2024). That study identified the optimal metal oxide loading for enhanced removal performance. The membrane prepared with formic acid achieved only 23% removal efficiency, while the Act:TFA combination yielded a 62% removal. The membrane synthesized using HFIP exhibited the highest efficiency at 97% in contrast to the pristine polyamide6 nanofiber, which demonstrated only 4.6% removal. All experiments were performed at an initial concentration of 10 mg/L, with adsorption measured at 1,440 min.

### 3.4 Phosphorus adsorption kinetics

**FIGURE 6:**
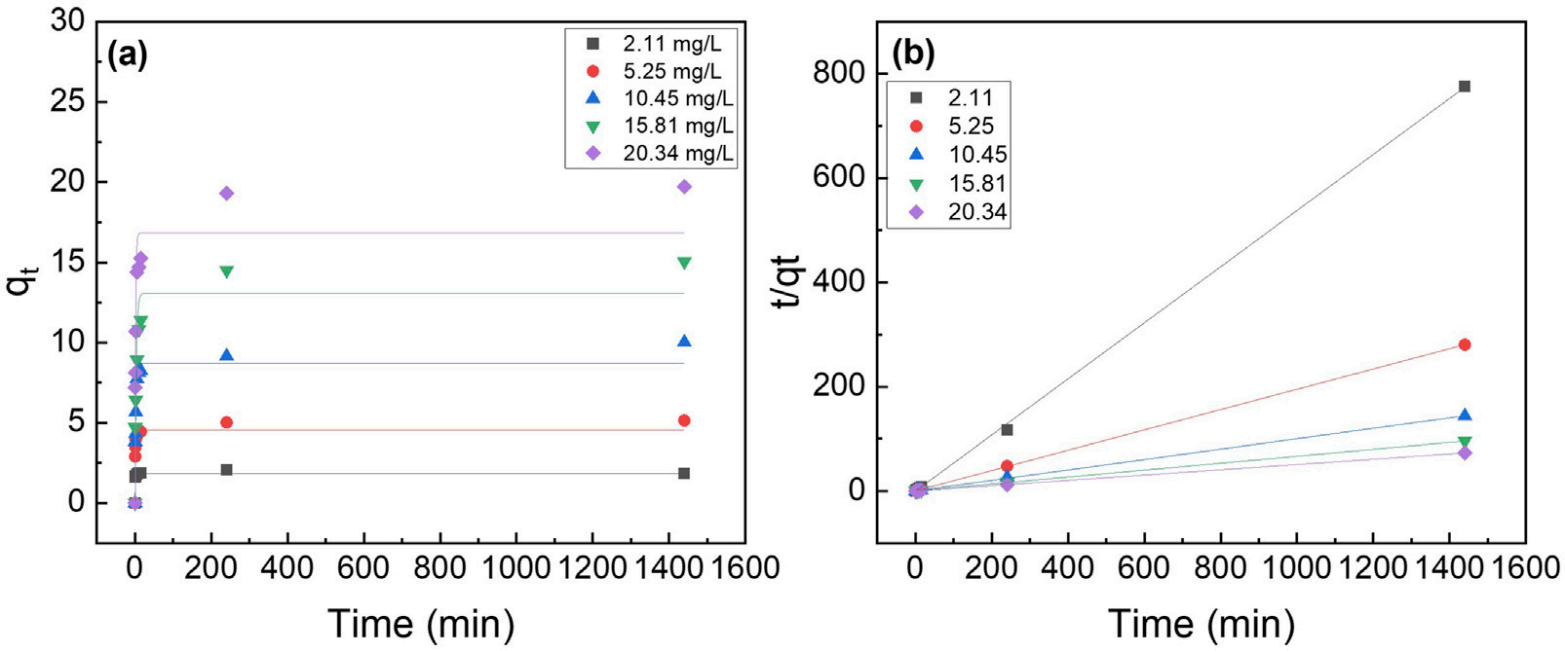
Effect of initial phosphate concentrations on the adsorption kinetics of PA6/La_2_O_3_/TBAB NF membrane at 23^°^C. Lines represent modeled fitting results using the **(a)** pseudo-first order and **(b)** pseudo second order equations. Adsorbent dosage: 1 g of La_2_O_3_/L.

The two most popular kinetic models, the pseudo-first-order and pseudo-second-order models (Equations 2, 3, respectively) were utilized to analyze the data. Figure 6 shows the pseudo-second order model fittings of suspended La_2_O_3_ nanoparticle and La_2_O_3_ composite NF membranes with various initial phosphate concentrations (i.e., 2, 5, 10, 15 and 20 mg/L). Table 1 shows the summarized kinetic correlation coefficient from the fitted data using Equations 2, 3. Based on the resulting correlation coefficients (i.e., R^2^ values), it can be concluded that La_2_O_3_ NP and composite NF membrane are best described by a pseudo-second-order kinetic model, which suggests chemisorption via ligand exchange of negatively charged phosphate ions with the lanthanum oxide surface (Kajjumba and Marti, 2022; He et al., 2022; Fang et al., 2018).

**TABLE 1:** The parameters for the pseudo first order and pseudo second order and the correlation coefficients. Adsorbent: 1 g/L (40 mg); Concentration: 10 (mg/L).

Sample	Pseudo first order			Pseudo second order		
	q_e_ (mg/g)	k_1_ (1/s)	R^2^	q_e_ (mg/g)	k_2_ (g/(mg · min))	R^2^
La_2_O_3_ NP	10.1	6.46E-02	0.99	10.4	1.60E-02	1.00
PLT NF	9.01	1.81E+00	0.92	10.0	2.63E-02	1.00
PLT NF 120°C	9.38	2.71E+00	0.96	10.25	1.49E-01	1.00
PLT NF 200°C	7.54	3.70E-03	0.92	7.75	2.24E-03	0.98

### 3.5 Phosphorus adsorption isotherms

**FIGURE 7:**
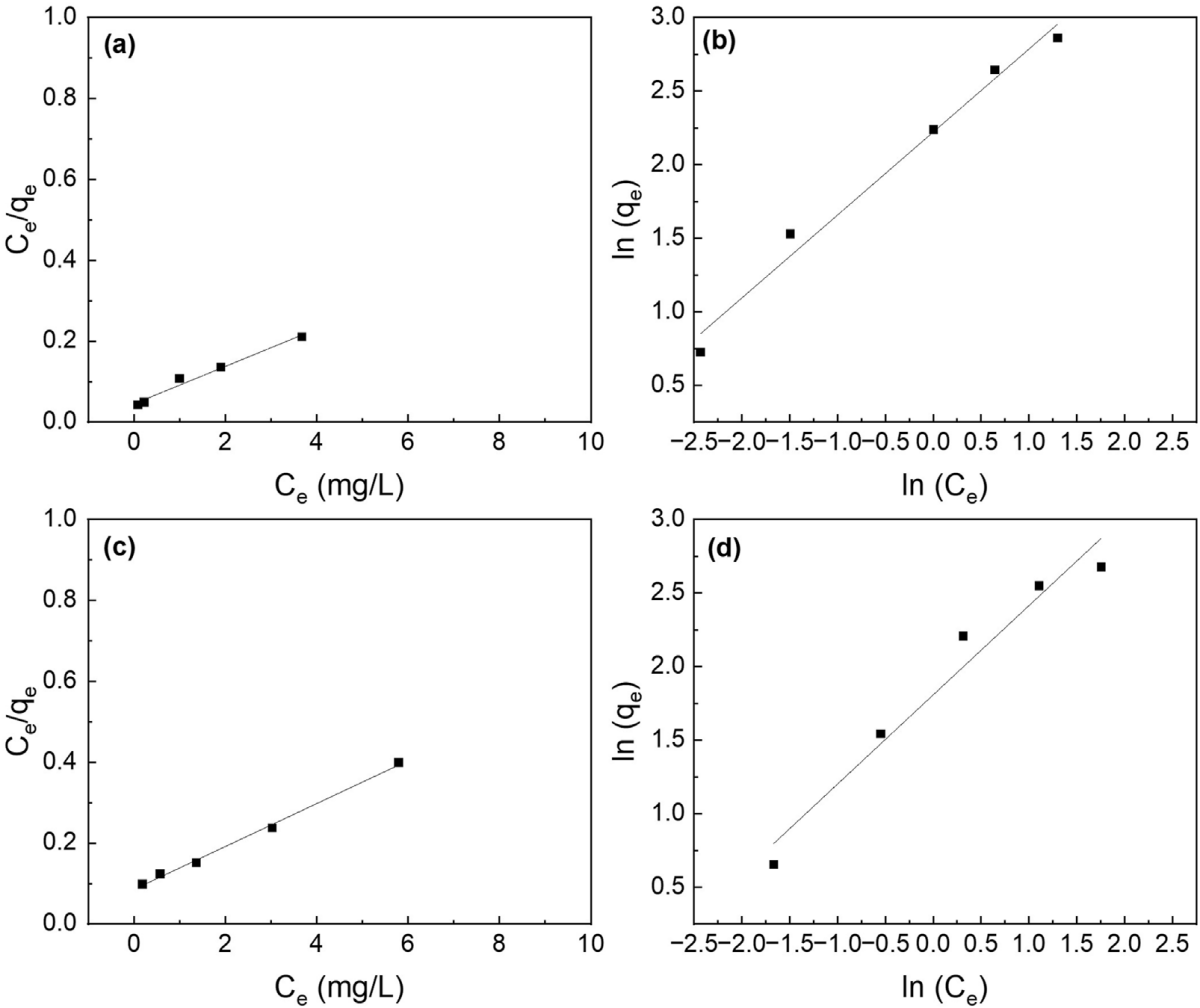
Effect of initial phosphate concentrations on the adsorption isotherms of **(a,b)** La_2_O_3_ NP, and **(c,d)** PA6/La_2_O_3_/TBAB NF membrane. Lines represent modeled fitting results using the **(a,c)** Langmuir isotherm and **(b,d)** Freundlich isotherm equations. Adsorbent dosage: 1 g/L.

The different adsorption isotherm models (i.e., Langmuir and Freundlich) were fitted to understand the adsorption process. The Langmuir isotherm model fits better than the Freundlich isotherm model (Figure 7). The maximum adsorption capacity (q_max_) for the composite nanofiber membrane was determined to be 18.8 mg P/mg which is slightly less than the suspended nanoparticles (21.6 mg P/g) (Table 2).

**TABLE 2:** The parameters for the Langmuir and Freundlich isotherms and the correlation coefficients for the phosphate adsorption. Adsorbent: 1 g/L (40 mg).

Sample	Langmuir isotherm			Freundlich isotherm		
	q_max_ (mg P/g)	K_L_ (L/mg)	R^2^	n	k_F_ (mg/g) · (L/mg)^1/n^	R^2^
La_2_O_3_ NP	21.6	1.03	0.98	1.77	9.22	0.97
PLT NF	18.8	0.622	0.99	1.65	6.09	0.95
PLT NF 120°C	17.4	1.02	0.94	1.86	7.22	0.92
PLT NF 200°C	17.3	0.469	0.86	1.81	4.98	0.97

### 3.6 Enhancing mechanical durability via post thermal treatment

**FIGURE 8:**
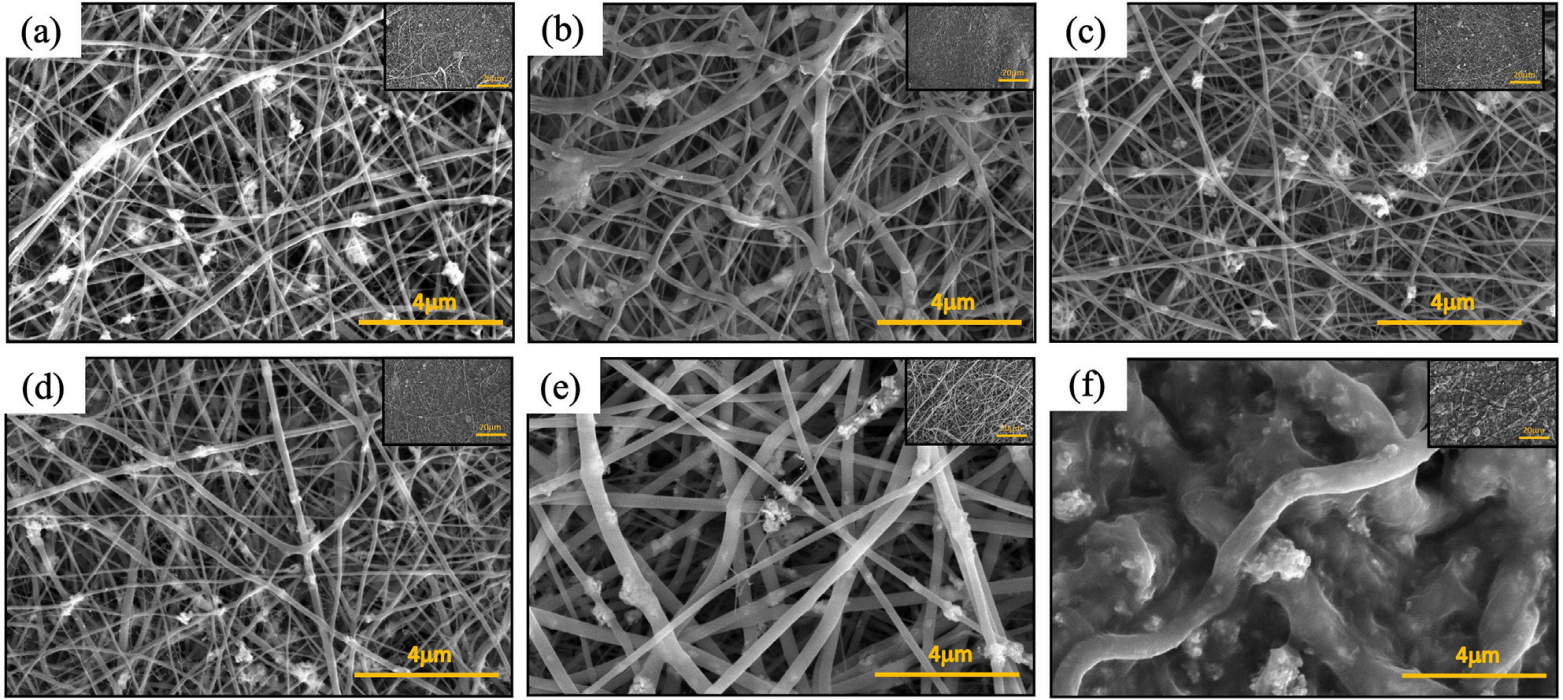
SEM images of PA6/La_2_O_3_/TBAB composite nanofibers: **(a)** as spun, and annealed at various temperatures: **(b)** 80°C, **(c)** 100°C, **(d)** 120°C, **(e)** 140°C, and **(f)** 200°C.

Polyamide exhibits two main crystalline structures, the α and γ phases (Zhang et al., 2011). The mechanical properties such as Young’s modulus are known to be higher for the α phase than for the γ phase (Liu et al., 2007; Dasgupta et al., 1996). While electrospun PA6 nanofibers predominately exist in the γ phase, the γ phase can transform into the α phase through post treatments, such as thermal processes (Ibanes et al., 2006; Park et al., 2005; Lincoln et al., 2004; Lincoln et al., 2001; Murthy et al., 1985; Carrizales et al., 2008; Dersch et al., 2003; Wang et al., 2014; Sanchaniya et al., 2023). During the post thermal process, the polymer chains are further aligned, leading to higher chain orientation, which has shown an increase in tensile strength in previous studies (Sheng et al., 2016; Ramaswamy et al., 2011; Srithep et al., 2013). SEM images of the tri-composite nanofiber membrane as-spun and post-thermally treated at various temperatures (i.e., 80, 100, 120, 140, and 200°C) are shown in Figure 8. As-spun nanofibers (Figure 8a) and samples annealed at various temperatures up to 120°C (Figures 8b–d) appear to remain similar in morphology, including the average fiber diameter (Supplementary Figures S4a–d). The sample annealed at 140°C (Figure 8e) showed nanofibers with a larger fiber diameter that have been measured to have an average fiber diameter of 240 nm (Supplementary Figure S4e) which is almost double that of the as-spun nanofibers (144 nm). Even with the increase in average fiber diameter at 140°C, optical images show no significant changes in color or shape of the nanofibers (Supplementary Figures S3a–e). When the samples were annealed at 200°C, the nanofiber color changed from white to tan (Supplementary Figure S3f) and shrunk in size. Additionally, the sample annealed at 200°C was rigid and brittle to touch compared to samples annealed at other temperatures, which remained flexible. Figure 8f shows the corresponding SEM image, which reveals deformation of nanofibers with an increased fiber diameters at 481 nm (Supplementary Figure S4f).

**FIGURE 9:**
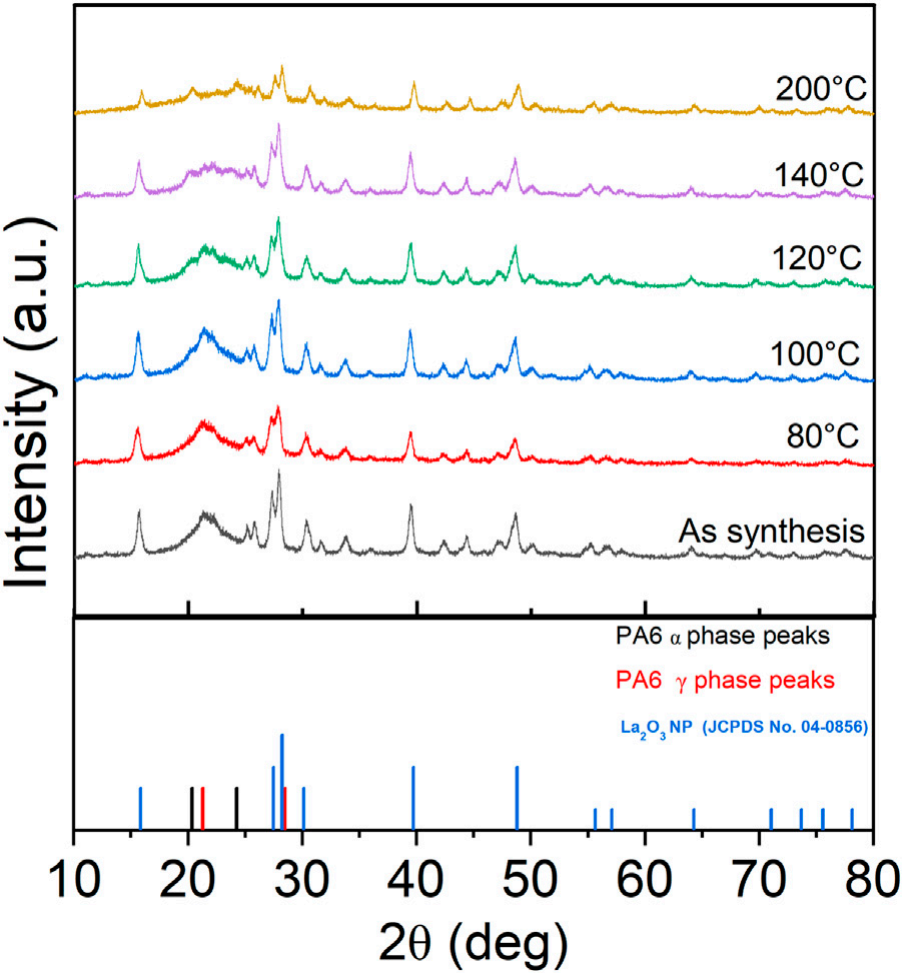
XRD analysis of tri-composite NF post thermally treated at various temperatures that shows changes in alpha and gamma phases.

**FIGURE 10:**
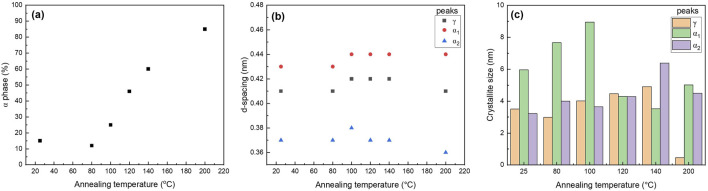
Graphs of **(a)** α phase content, **(b)** d-spacing, and **(c)** crystallite size of PA6 as a function of various annealing temperatures.

XRD patterns show the presence of the γ phase (peak at 21.3°) in pristine nanofiber, with an increase in diffraction intensity as the sample was thermally annealed up to 100°C (Figure 9). When the post thermal treatment temperature was increased above 100°C, two additional peaks started to appear around 20.5° and 24.4° which are corresponding to the (200) and (002)/(202) peaks of the α phase (Liu et al., 2007; Zhang et al., 2011). As the annealing temperature was further increased to 120°C and 140°C, the γ phase transformed to the thermally stable α phase which was shown by decreased intensity of the peak present at 21.3°. The sample that was annealed at 200°C showed two distinct α phase peaks and muted γ phase peak. The α and γ phase percentages have been calculated via peak deconvolution using OriginPro and the results are summarized in Table 3. Figure 10a shows that when the sample was annealed at 80°C, the α phase percentage stayed around 12%–15%. Further increase in annealing temperature from 100 to 200°C significantly increased the content up to 85%. D-spacing and crystallite size for α and γ phase peaks were tabulated in Table 3. The d-spacing can vary depending on the annealing temperature and the introduction of additives due to changes in thermal expansion and contraction which affect the length of the hydrogen bond (Ramesh, 1999; Radusch et al., 1994). Calculated d-spacing for α phase peaks were ∼0.43 and ∼0.37 nm for (200) and mixed (002)/(202), respectively. The d-spacing for the γ phase peak was determined to be ∼0.41 nm. In the α phase, the d-spacing of the (200) plane represents the interchain distance within the sheet and the d-spacing of the (002)/(202) plane represents the inter-sheet distance between the sheets. When a peak is shifted to a higher angle, it represents a decrease in the hydrogen bond length which translates to a decrease in d-spacing. Conversely, a peak shift to a lower angle represents an increase in hydrogen bond length due to thermal expansion and an increase in d-spacing (Zhang et al., 2011). Figure 10b shows a trend of increasing d-spacing values for α_1_ and γ peaks at 100°C, maintaining this value with a plateau or decrease in d-spacing values at 200°C, respectively. α_2_ peak showed an increase in d-spacing value at 100°C but then showed a gradual decrease as the annealing temperature increased to 200°C. While the composite nanofiber membrane were synthesized using different method and has a different composition compared to pristine polyamide 6 films, or nonwoven fibers, the d-spacing values obtained are comparable to previously reported polyamide 6 materials (Liu et al., 2007; Ramesh, 1999; Cho et al., 2012; Ramesh et al., 1994; King, 2004).

**TABLE 3:** Peak location and d-spacing based on XRD on PA6/La_2_O_3_/TBAB composite NF annealed at various temperatures.

Annealing temp (°C)	2ө (°)	Crystallite size (nm)	d-spacing (nm)	Area integrated (%)
Pristine	γ: 21.4	3.51	0.41	85
	α: 20.53	5.96	0.43	15
	α: 24.33	3.22	0.37	
80	γ: 21.55	2.98	0.41	88
	α: 20.42	7.66	0.43	12
	α: 24.08	4.00	0.37	
100	γ: 21.33	4.01	0.42	75
	α: 20.37	8.95	0.44	25
	α: 24.41	3.65	0.38	
120	γ: 21.36	4.46	0.42	54
	α: 20.32	4.29	0.44	46
	α: 24.36	4.28	0.37	
140	γ: 21.39	4.91	0.42	40
	α: 20.37	3.52	0.44	60
	α: 24.29	6.38	0.37	
200	γ: 21.52	0.46	0.41	15
	α: 20.30	5.02	0.44	85
	α: 24.48	4.50	0.36	

In general, the crystallinity of the α phase-dominated PA6 increased during post thermal annealing (Hindeleh and Johnson, 1978; Vasanthan et al., 2009). When there is a decrease in peak width, the crystallite size increases in the direction of the hydrogen bond due to improvement in lattice order (Hindeleh and Johnson, 1978; Vasanthan et al., 2009). Figure 10c shows the changes in PLT NF crystallite size as a function of annealing temperature. The graphs show that there is an initial dip in the crystallite size of the γ-phase up to 80°C, and then a continuous increase until 200°C. The significant decrease in γ-phase crystallite size at 200°C may be attributed to the meta-stable γ- phase transitioning into thermodynamically stable α-phase at higher temperature through melting and recrystallization (Liu et al., 2007; Lincoln et al., 2001).

**FIGURE 11:**
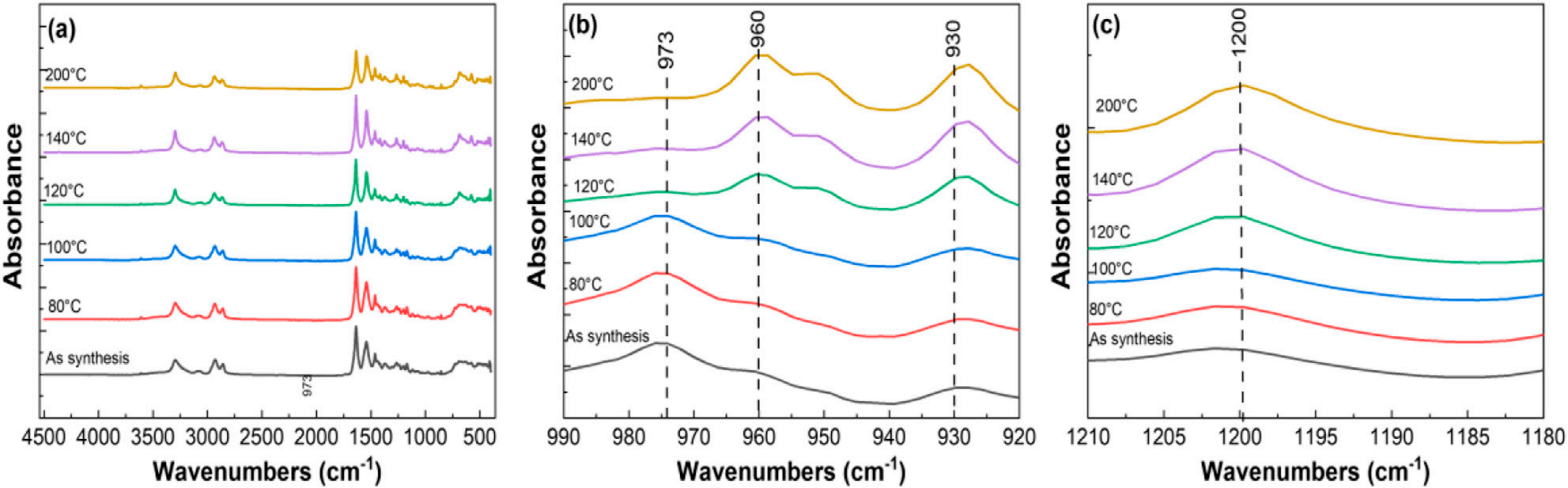
FTIR analysis of tri-composite NF post thermally treated at various temperatures in the ranges of **(a)** 450–4,500 cm^−1^, **(b)** 920–990 cm^−1^, and **(c)** 1,190–1,210 cm^−1^.

FTIR spectra for various tri-composite samples annealed at different temperatures are shown in Figure 11a, spanning the range from 450 to 4,500 cm^−1^. Polyamide 6 typically exist in α- and γ- crystalline forms. The α-phase consists of sheets of fully extended planar chains joined by the hydrogen bonds between antiparallel chains. In contrast, γ- phase comprises sheets of parallel chains linked by hydrogen bond between the adjacent chains (Arimoto et al., 1965). Figure 11b presents an enlarged view focusing on the characteristic polyamide6 peaks between 920 and 990 cm^−1^. The bands at 930, and 960 cm^−1^ are attributed to the α- phase, while the band at 973 cm^−1^ corresponds to the γ-phase (Porubská et al., 2012; Miri et al., 2011; Vasanthan and Salem, 2001). As the annealing temperature increases, the emergence and sharpening of peaks at 930 and 960 cm^−1^ indicates a transition to a fully planar conformation, representing transformation to the α- phase. Conversely, the gradual disappearance of the peak at 973 cm^−1^reflects the conversion of the γ-phase into α- phase. Figure 11c shows peak at 1,200 cm^−1^—assigned to c-c stretching—is present in both phases but appears sharper in the α-phase due to its higher crystallinity and denser hydrogen bonding compared to the γ-phase.

**FIGURE 12:**
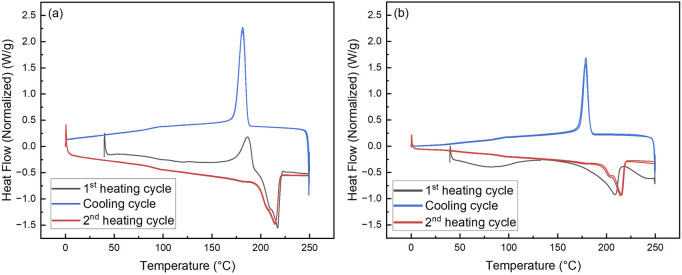
Differential scanning calorimetry (DSC) of **(a)** PA6 NF and **(b)** PA6/La_2_O_3_/TBAB composite NF.

Differential scanning calorimetry (DSC) was used to examine the melting and crystallization behaviors of electrospun pristine PA6 nanofiber mat and PA6/La_2_O_3_/TBAB composite nanofiber mat. The glass transition temperature (T_g_), crystallization temperature (T_c_), and melting temperature (T_m_) of the two samples are summarized in Table 4. Figure 12 shows first heating, cooling cycle, and reheating cycles of the samples. The glass transition temperature for the composite nanofiber was calculated to be lower than pristine nanofiber, decreasing from 89.8°C to 68.5°C. This could be due to adding lanthanum oxide nanoparticles and TBAB, which adds defects to electrospun polymeric nanofibers (Zhang et al., 2011; Sridhara et al., 2021). The distinct exothermic crystallization peak shown at 186.8 C for electrospun PA6 nanofiber membrane that typically exist in γ-phase can refer to recrystallization to thermostable α-phase. The melting endotherm occurring with a peak temperature T_m_ ∼ 217.4°C has a shoulder peak around 200°C and these two peaks can correspond to α- and γ-phase which is typical for samples that have multiple phases (Su et al., 2007; Khanna and Kuhn, 1997). The DSC graph of composite nanofiber mat shows a very small and short exothermic crystallization peak around 132.8°C, followed by two broad endothermic melting peaks. When the composite nanofiber was annealed at various temperatures, XRD data showed an increase in alpha phase peak ratios (Figure 10a) as the calculated glass transition temperature (∼68.5°C) exceeded which can be correlated with the exothermic peak that was shown in Figure 12b. In the first heating cycle, a distinct melting peak is shown around 209.2°C followed by smaller melting peak around 250°C The two melting peaks can correspond to melting of γ peak and then thermostable α peaks, respectively. The XRD in Figure 9 shows disappearance of γ peak at 200 °C and it can be assumed that the melting peak shown at 250°C on DSC data (Figure 12b) corresponds to the remaining α-phase. While the first heating cycle shows two melting peaks at 209.2°C and 250°C, the reheating cycle shows two peaks joint, one main and one as a shoulder endothermic melting peak. While the first heating cycle takes the processing and environmental condition into account, the reheating cycle shows the melting behavior that is correlated to controlled and accurate analysis of the sample as the thermal history is erased by the cooling cycle (Mileva et al., 2012). While the electrospun composite nanofiber showed two distinct melting peaks in the first heating cycle, when reheated, it exhibits similar one endothermic melting peak like pristine PA6 NF.

**TABLE 4:** Thermal parameters for the electrospun PA6 NF and PA6/La_2_O_3_/TBAB NF.

Sample	T_g_ (°C)	T_c_ (°C)	T_m_ (°C)
PA6 NF	89.8	186.8	217.4
PA6/La_2_O_3_/TBAB NF	68.5	132.8	209.2

**FIGURE 13:**
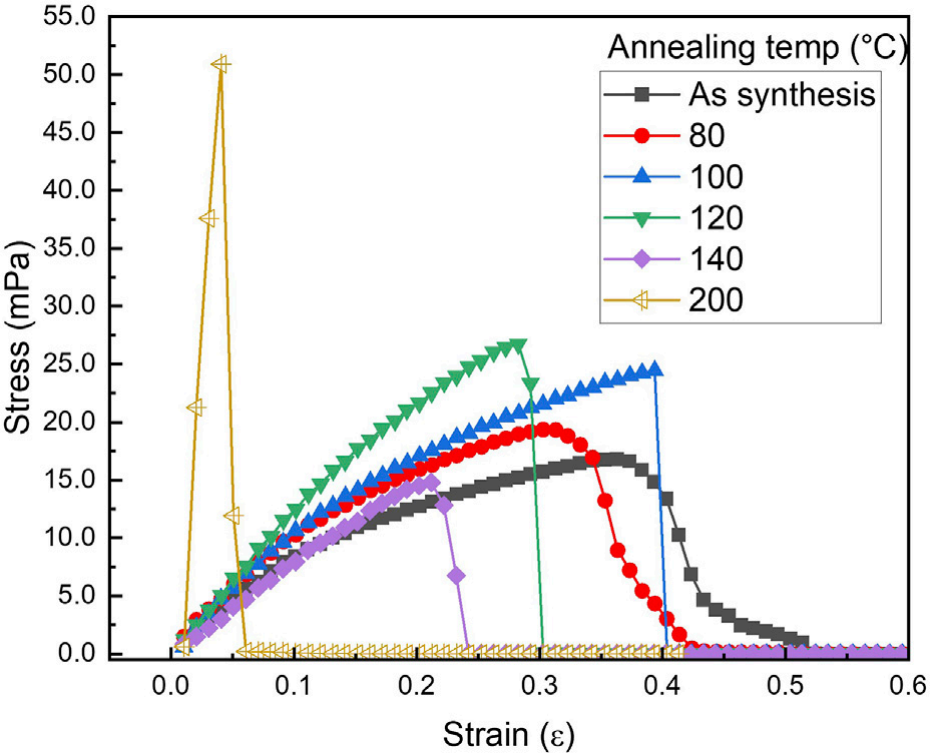
Stress vs. strain graph of PA6/La_2_O_3_/TBAB NF as-spun and annealed at various temperatures.

Figure 13 shows a stress vs. strain plot of PLT NF with different post thermal treatment. Various mechanical property parameters such as Young’s modulus, yield strength, ultimate tensile strength, toughness, and strain to fracture have been calculated and the obtained results are listed in Table 5. Young’s modulus initially decreased followed by an increase until 120°C. Further increases in annealing temperature resulted in a decrease in Young’s modulus. While the Young’s modulus for PLT NF annealed at 200°C showed the highest value, this sample lost flexibility and mechanical durability (Su et al., 2007). From this point on, PLT NF annealed at 200°C is excluded from comparing mechanical durability. Yield strength, which is the maximum stress that can be withstood before permanent deformation, showed its maximum value when the sample was annealed at 120°C.

**TABLE 5:** Mechanical properties of PA6/La_2_O_3_/TBAB composite NF calculated from the stress vs. strain graph at different annealing temperatures.

Annealing temperature (°C)	Young’s modulus (Pa)	Yield strength (Pa)	Ultimate tensile strength (Pa)	Toughness (J·m^−3^)	Strain to fracture (ε)
As synthesis	7.78E+08	1.09E+07	1.68E+07	5.07E+06	3.63E-01
80	8.90E+07	1.45E+07	1.94E+07	5.01E+06	3.13E-01
100	9.68E+07	1.36E+07	2.44E+07	6.17E+06	3.93E-01
120	1.26E+08	1.46E+07	2.67E+07	4.74E+06	2.82E-01
140	7.94E+07	9.76E+06	1.35E+07	1.76E+06	2.32E-01
200	1.83E+09	3.76E+07	5.09E+07	1.22E+06	5.07E-02

**FIGURE 14:**
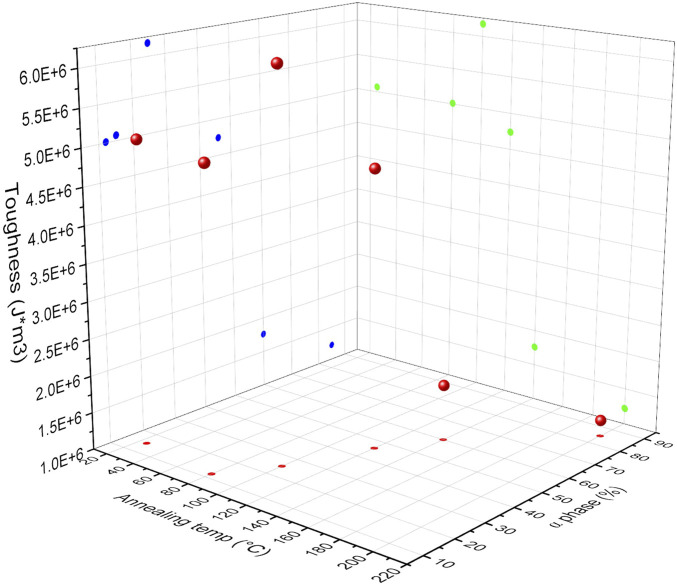
3D plot of toughness of composite membrane as function of annealing temperature and α phase %.

Figure 14 shows a 3D plot of toughness of the material as a function of annealing temperature and calculated α-phase content. Results show that with an increase in annealing temperature and α-phase percentage, an initial increase and then decrease in toughness of the material is observed. The relationship between annealing temperature and calculated α-phase content is represented in red, toughness as a function of annealing temperature in green, and toughness as a function of calculated α-phase content in blue. These three graphs are combined into a 3D representation, illustrating the interdependence of toughness, annealing temperature, and calculated α-phase content. The 3D graph shows that the toughness of the material reached its maximum when samples were annealed at 100°C, with an α-phase content of 25%. Although the mechanical properties of polyamide 6 were more closely correlated to the α phase, PLT NF annealed at 200°C, which had the highest α phase content, showed poor mechanical properties due to the degradation of the polymer, which resulted in the brittleness of the sample. This indicates that although PLT NF annealed at 200°C had the highest α-phase percentage, this sample was not mechanically durable enough to be used as a filtration membrane. In addition to toughness of the material, PLT NF showed maximized Young’s modulus, Yield strength, and ultimate tensile strength when the sample was annealed at 120°C. Based on this observation, PLT NF annealed at 120°C was deemed suitable for optimization in mechanical durability through the post thermal process.

### 3.7 Effect of post thermal treatment on phosphate removal

**FIGURE 15:**
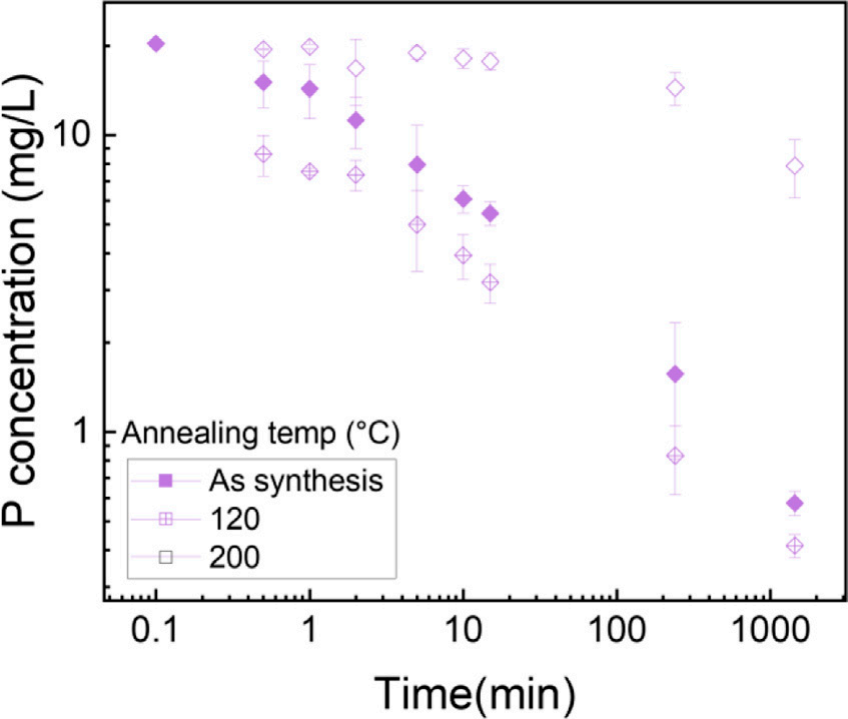
Log-log plot of phosphate uptake as function of time using PA6/La_2_O_3_/TBAB NF annealed at various temperatures. Initial phosphate concentration: 20.34 mg/L, adsorbent dosage 1 g/L.

**FIGURE 16:**
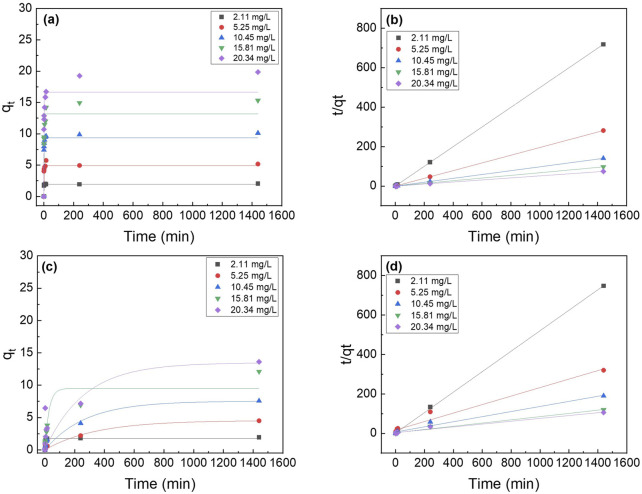
Effect of initial phosphate concentrations on the adsorption kinetics of PA6/La_2_O_3_/TBAB NF membrane annealed at various temperatures: **(a,b)** 120°C and **(c,d)** 200°C. Lines represent modeled fitting results using the **(a,c)** pseudo-first order and **(b,d)** pseudo second order equations. Adsorbent dosage: 1 g/L.

Phosphorus batch testing was also performed to understand the effect of post thermal processing on the adsorption capacity of the membrane. Figure 15 shows a log-log plot of phosphate uptake of various annealed samples with a fixed initial concentration of P at 20.34 mg/L. It shows that the adsorption of PLT NF annealed at 120°C had higher P removal and faster kinetics compared to as-spun PLT NF and PLT NF annealed at 200°C. The decreased phosphorous adsorption observed from the sample heat treated at 200°C could be due to deformed structure of the electrospun nanofiber membrane. As seen in Figure 8f, the SEM image shows deformed nanofibers that are not porous compared to the rest of the samples (Figures 8a–e). The structural deformation can lead to a reduction in specific surface area and porosity, which directly impacts adsorption efficiency (Cui et al., 2020; Zhu et al., 2021; Wang and Hsiao, 2016; HMTShirazi et al., 2022). Figure 16 shows pseudo first-order and pseudo-second-order models fitting of PLT NF annealed at 120°C and PLT NF annealed at 200°C. Table 1 lists the obtained kinetic data. PLT NF annealed at 120°C and 200°C followed pseudo-second-order kinetics like as-spun composite nanofibers. Additionally, PLT NF annealed at 120°C was faster compared to the rest of the samples at 1.49E^-^01 g/(mg·min).

**FIGURE 17:**
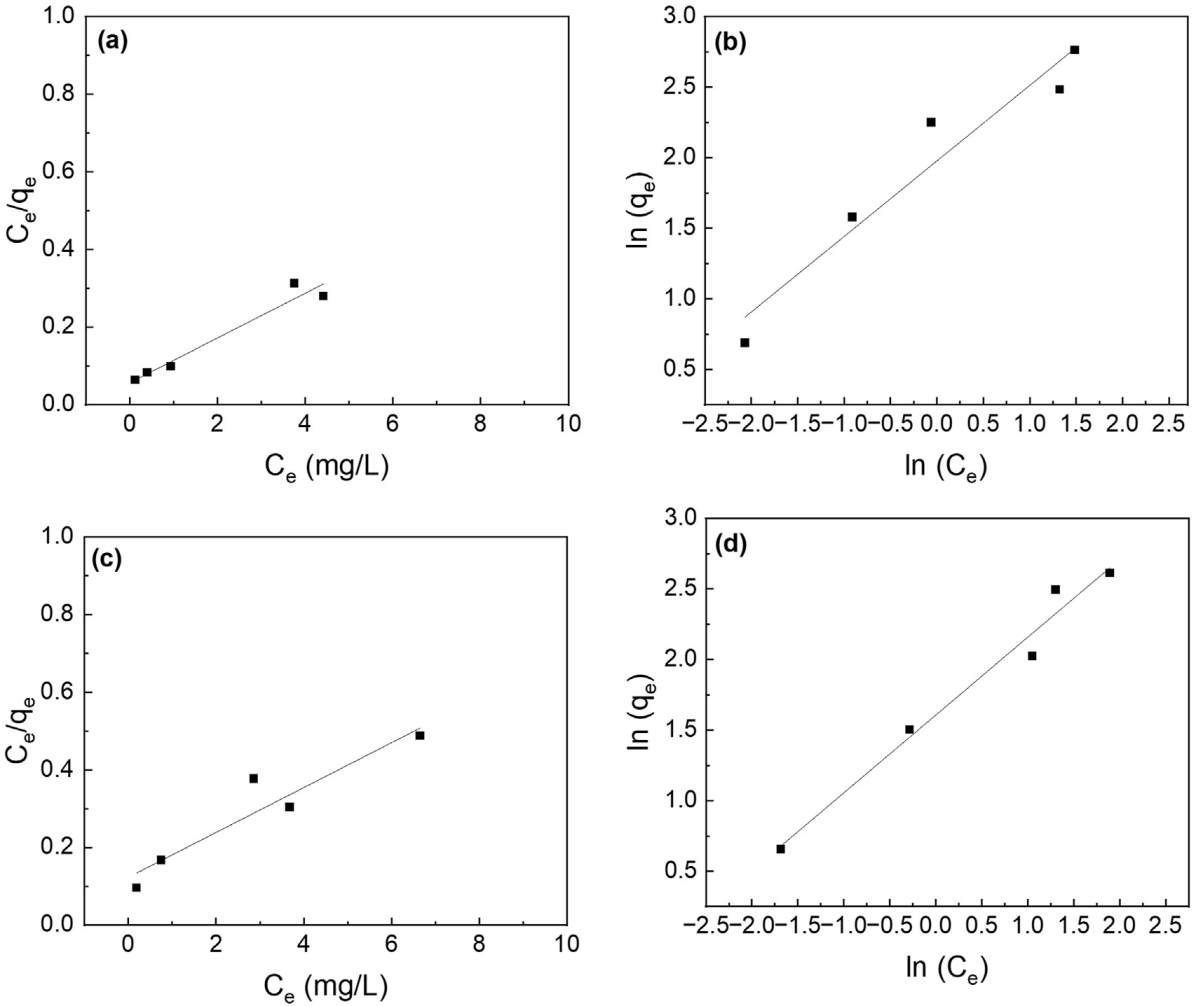
Effect of initial phosphate concentrations on the adsorption isotherm of PA6/La_2_O_3_/TBAB NF membrane annealed at various temperatures: **(a,b)** 120°C and **(c,d)** 200°C. Lines represent modeled fitting results using the **(a,c)** Langmuir isotherm and **(b,d)** Freundlich isotherm equations. Adsorbent dosage: 1 g/L.

Figure 17 shows the fitted curves using the Langmuir and Freundlich isotherm models, and the calculated adsorption capacity is listed in Table 2. The Langmuir isotherm model fits the experimental data better than Freundlich’s isotherm model for all the samples except for PLT NF annealed at 200°C. PLT NF annealed at 120°C showed ∼19.4% and ∼7.5% decreased in q_max,_ compared to suspended La_2_O_3_ NP and as-spun PLT nanofibers, respectively. The difference in q_max_ value may be attributed to the alteration of porosity and surface area during the post thermal treatment process. Despite the lower calculated q_max,_ value, PLT NF annealed at 120°C was able to remove 97% of P which is same as-spun PLT NF.

The reusability of polyamide nanofiber membrane has been investigated, with various studies reporting a decrease in adsorption efficiency during second to fifth re-adsorption cycles, likely due to the blockage of active sites. However, the adsorption performance remained relatively high, exceeding 80% against bisphenol (Jasni et al., 2017). Zarrini et al., demonstrated that dye removal using polyamide 6 nanofibers exhibited only a 10% reduction in adsorption capacity, confirming their viability as reusable adsorbents (Zarrini et al., 2017). Other studies have also shown that polyamide 6 based nanofibrous membranes maintain good reusability, with no significant changes observed even after five to eight reusability tests (Amaly et al., 2020; Kakoria et al., 2021).

## 4 Conclusion

Mechanically robust La_2_O_3_ nanoparticles embedded polyamide 6 nanofibers were synthesized using electrospinning followed by post thermal treatment. Out of three solvents (i.e., formic acid, Ace:TFA mixture, and HFIP), HFIP allowed colloidal suspension of La_2_O_3_ nanoparticles. TEM and powder XRD patterns confirmed that La_2_O_3_ nanoparticles embedded in PA6 nanofiber without changing their composition. Batch phosphorus removal testing showed that the composite nanofiber showed similar P removal efficiency as suspended nanoparticles. Post thermal treatment was utilized to tune the crystal phases of the composite nanofiber membrane to enhance the mechanical properties where the crystal structure of electrospun nanofibers changed from γ-phase to α-phase with an increase in post thermal treatment temperature. Samples annealed at 120°C showed optimized yield strength and ultimate tensile strength with an increased kinetic constant rate while maintaining adsorption capacity.

## Data Availability

The raw data supporting the conclusions of this article will be made available by the authors, without undue reservation.

## References

[B1] Ahmad J. Zaid O. Aslam F. Martínez-García R. Alharthi Y. M. Hechmi Ei Ouni M. (2021). Mechanical properties and durability assessment of Nylon fiber reinforced self-compacting concrete. J. Eng. Fibers Fabr. 16, 15589250211062833. 10.1177/15589250211062833

[B2] Ajmal Z. Muhmood A. Usman M. Kizito S. Lu J. Dong R. (2018). Phosphate removal from aqueous solution using iron oxides: adsorption, desorption and regeneration characteristics. J. colloid interface Sci. 528, 145–155. 10.1016/j.jcis.2018.05.084 29843062

[B3] Amaly N. El-Moghazy A. Y. Si Y. Sun G. (2020). Functionalized nanofibrous Nylon 6 membranes for efficient reusable and selective separation of laccase enzyme. Colloids Surfaces B Biointerfaces 194, 111190. 10.1016/j.colsurfb.2020.111190 32593859

[B4] Arimoto H. Ishibashi M. Hirai M. Chatani Y. (1965). Crystal structure of the γ-form of Nylon 6. J. Polym. Sci. Part A General Pap. 3 (1), 317–326. 10.1002/pol.1965.100030132

[B5] Aziz S. Mazhar A. R. Ubaid A. Shah S. M. H. Riaz Y. Talha T. (2024). A comprehensive review of membrane-based water filtration techniques. Appl. Water Sci. 14 (8), 169. 10.1007/s13201-024-02226-y

[B6] Bunce J. T. Ndam E. Ofiteru I. D. Moore A. Graham D. W. (2018). A review of phosphorus removal technologies and their applicability to small-scale domestic wastewater treatment systems. Front. Environ. Sci. 6. 10.3389/fenvs.2018.00008

[B7] Carrizales C. Pelfrey S. Rincon R. Eubanks T. M. Kuang A. McClure M. J. (2008). Thermal and mechanical properties of electrospun PMMA, PVC, Nylon 6, and Nylon 6,6. Polym. Adv. Technol. 19 (2), 124–130. 10.1002/pat.981

[B8] Cho H.-H. Lee M.-H. Kim G.-Y. Lee S.-H. Noh H.-K. (2012). Three directional crystal structure changes of Nylon 6-ran-Nylon 4 copolymer films by drawing ratio and elevating temperature. Text. Sci. Eng. 49 (2), 106–111. 10.12772/TSE.2012.49.2.106

[B9] Choi Y. Y. Hanh To D. T. Kim S. Cwiertny D. M. Myung N. V. (2024). Mechanically durable tri-composite polyamide 6/hematite nanoparticle/tetra-n-butylammonium bromide (PA6/α-Fe2O3/TBAB) nanofiber based membranes for phosphate remediation. Front. Chem. 12, 1472640. 10.3389/fchem.2024.1472640 39314992 PMC11416959

[B10] Cui J. Li F. Wang Y. Zhang Q. Ma W. Huang C. (2020). Electrospun nanofiber membranes for wastewater treatment applications. Sep. Purif. Technol. 250, 117116. 10.1016/j.seppur.2020.117116

[B11] Dasgupta S. Hammond W. B. Goddard W. A. (1996). Crystal structures and properties of Nylon polymers from theory. J. Am. Chem. Soc. 118 (49), 12291–12301. 10.1021/ja944125d

[B12] Dersch R. Liu T. Schaper A. K. Greiner A. Wendorff J. H. (2003). Electrospun nanofibers: internal structure and intrinsic orientation. J. Polym. Sci. Part A Polym. Chem. 41 (4), 545–553. 10.1002/pola.10609

[B13] De Schoenmaker B. Van der Heijden S. De Baere I. Van Paepegem W. De Clerck K. (2013). Effect of electrospun polyamide 6 nanofibres on the mechanical properties of a glass fibre/epoxy composite. Polym. Test. 32 (8), 1495–1501. 10.1016/j.polymertesting.2013.09.015

[B14] Ding D. Li Z. Yu S. Yang B. Yin Y. Zan L. (2022). Piezo-photocatalytic flexible PAN/TiO2 composite nanofibers for environmental remediation. Sci. Total Environ. 824, 153790. 10.1016/j.scitotenv.2022.153790 35150683

[B15] Durán-Sánchez A. Álvarez-García J. Del Río-Rama M. D. la C. (2018). Sustainable water resources management: a bibliometric overview. Water 10 (9), 1191. 10.3390/w10091191

[B16] Fang L. Wu B. Chan J. K. M. Lo I. M. C. (2018). Lanthanum oxide nanorods for enhanced phosphate removal from sewage: a response surface methodology study. Chemosphere 192, 209–216. 10.1016/j.chemosphere.2017.10.154 29102865

[B17] Frontiers (2025). A review of phosphorus removal technologies and their applicability to small-scale domestic wastewater treatment systems. Available online at: https://www.frontiersin.org/journals/environmental-science/articles/10.3389/fenvs.2018.00008/full (Accessed January 29, 2025).

[B18] Ge X. Chen X. Liu M. Wang C. Zhang Y. Wang Y. (2023). Toward a better understanding of phosphorus nonpoint source pollution from soil to water and the application of amendment materials: research trends. Water 15 (8), 1531. 10.3390/w15081531

[B19] Greenstein K. E. Myung N. V. Parkin G. F. Cwiertny D. M. (2019). Performance comparison of hematite (α-Fe2O3)-polymer composite and core-shell nanofibers as point-of-use filtration platforms for metal sequestration. Water Res. 148, 492–503. 10.1016/j.watres.2018.10.048 30408735

[B20] Guibo Y. Qing Z. Yahong Z. Yin Y. Yumin Y. (2013). The electrospun polyamide 6 nanofiber membranes used as high efficiency filter materials: filtration potential, thermal treatment, and their continuous production. J. Appl. Polym. Sci. 128 (2), 1061–1069. 10.1002/app.38211

[B21] Guida S. Rubertelli G. Jefferson B. Soares A. (2021). Demonstration of ion exchange technology for phosphorus removal and recovery from municipal wastewater. Chem. Eng. J. 420, 129913. 10.1016/j.cej.2021.129913

[B22] He Q. Zhao H. Teng Z. Wang Y. Li M. Hoffmann M. R. (2022). Phosphate removal and recovery by lanthanum-based adsorbents: a review for current advances. Chemosphere 303, 134987. 10.1016/j.chemosphere.2022.134987 35597457

[B23] Heikkilä P. Harlin A. (2008). Parameter study of electrospinning of polyamide-6. Eur. Polym. J. 44 (10), 3067–3079. 10.1016/j.eurpolymj.2008.06.032

[B24] Hindeleh A. M. Johnson D. J. (1978). Crystallinity and crystallite size measurement in polyamide and polyester fibres. Polymer 19 (1), 27–32. 10.1016/0032-3861(78)90167-2

[B25] Hmtshirazi R. Mohammadi T. Asadi A. A. Tofighy M. A. (2022). Electrospun nanofiber affinity membranes for water treatment applications: a review. J. Water Process Eng. 47, 102795. 10.1016/j.jwpe.2022.102795

[B26] Ibanes C. de Boissieu M. David L. Seguela R. (2006). High temperature behaviour of the crystalline phases in unfilled and clay-filled Nylon 6 fibers. Polymer 47 (14), 5071–5079. 10.1016/j.polymer.2006.05.025

[B72] Izadi P. Izadi P. Eldyasti S. (2020). Design, operation and technology configurations for enhanced biological phosphorus removal (EBPR) process: a review. Rev. Environ. Sci. Biotechnol. 19, 561–593. 10.1007/s11157-020-09538-w

[B27] Jasni M. J. F. Arulkumar M. Sathishkumar P. Mohd Yusoff A. R. Buang N. A. Gu F. L. (2017). Electrospun Nylon 6,6 membrane as a reusable nano-adsorbent for bisphenol A removal: adsorption performance and mechanism. J. Colloid Interface Sci. 508, 591–602. 10.1016/j.jcis.2017.08.075 28869916

[B28] Kajjumba G. W. Marti E. J. (2022). A review of the application of cerium and lanthanum in phosphorus removal during wastewater treatment: characteristics, mechanism, and recovery. Chemosphere 309, 136462. 10.1016/j.chemosphere.2022.136462 36162516

[B29] Kakoria A. Sinha-Ray S. Sinha-Ray S. (2021). Industrially scalable chitosan/nylon-6 (CS/N) nanofiber-based reusable adsorbent for efficient removal of heavy metal from water. Polymer 213, 123333. 10.1016/j.polymer.2020.123333

[B30] Khanna Y. P. Kuhn W. P. (1997). Measurement of crystalline index in nylons by DSC: complexities and recommendations. J. Polym. Sci. Part B Polym. Phys. 35 (14), 2219–2231. 10.1002/(SICI)1099-0488(199710)35:14<2219::AID-POLB3>3.0.CO;2-R

[B31] Kim J. Deng Q. Benjamin M. M. (2008). Simultaneous removal of phosphorus and foulants in a hybrid coagulation/membrane filtration system. Water Res. 42 (8), 2017–2024. 10.1016/j.watres.2007.12.017 18222523

[B32] King S. (2004). SANS from surfactant-treated Nylon fibres. FDR 12 (12), 41. 10.1382/s20041241

[B33] Lincoln D. M. Vaia R. A. Krishnamoorti R. (2004). Isothermal crystallization of nylon-6/montmorillonite nanocomposites. Macromolecules 37 (12), 4554–4561. 10.1021/ma049768k

[B34] Lincoln D. M. Vaia R. A. Wang Z.-G. Hsiao B. S. Krishnamoorti R. (2001). Temperature dependence of polymer crystalline morphology in Nylon 6/montmorillonite nanocomposites. Polymer 42 (25), 09975–09985. 10.1016/S0032-3861(01)00542-0

[B35] Liu Y. Cui L. Guan F. Gao Y. Hedin N. E. Zhu L. (2007). Crystalline morphology and polymorphic phase transitions in electrospun Nylon 6 nanofibers. Macromolecules 40 (17), 6283–6290. 10.1021/ma070039p 18698379 PMC2507769

[B36] Luo D. Wang L. Nan H. Cao Y. Wang H. Kumar T. V. (2023). Phosphorus adsorption by functionalized biochar: a review. Environ. Chem. Lett. 21 (1), 497–524. 10.1007/s10311-022-01519-5

[B37] Mileva D. Kolesov I. Androsch R. (2012). Morphology of cold-crystallized polyamide 6. Colloid Polym. Sci. 290 (10), 971–978. 10.1007/s00396-012-2657-3

[B38] Miri V. Persyn O. Seguela R. Lefebvre J. M. (2011). On the deformation induced order–disorder transitions in the crystalline phase of polyamide 6. Eur. Polym. J. 47 (1), 88–97. 10.1016/j.eurpolymj.2010.09.006

[B39] Murthy N. S. Aharoni S. M. Szollosi A. B. (1985). Stability of the γ form and the development of the α form in Nylon 6. J. Polym. Sci. Polym. Phys. Ed. 23 (12), 2549–2565. 10.1002/pol.1985.180231212

[B40] Nalbandian M. J. Kim S. Gonzalez-Ribot H. E. Myung N. V. Cwiertny D. M. (2022). Recent advances and remaining barriers to the development of electrospun nanofiber and nanofiber composites for point-of-use and point-of-entry water treatment systems. J. Hazard. Mater. Adv. 8, 100204. 10.1016/j.hazadv.2022.100204 37025391 PMC10074328

[B41] Othman A. Vargo P. Andreescu S. (2019). Recyclable adsorbents based on ceria nanostructures on mesoporous silica beads for the removal and recovery of phosphate from eutrophic waters. ACS Appl. Nano Mater. 2 (11), 7008–7018. 10.1021/acsanm.9b01512

[B42] Paerl H. W. Hall N. S. Calandrino E. S. (2011). Controlling harmful cyanobacterial blooms in a world experiencing anthropogenic and climatic-induced change. Sci. Total Environ. 409 (10), 1739–1745. 10.1016/j.scitotenv.2011.02.001 21345482

[B43] Park S.-Y. Cho Y.-H. Vaia R. A. (2005). Three-dimensional structure of the zone-drawn film of the nylon-6/layered silicate nanocomposites. Macromolecules 38 (5), 1729–1735. 10.1021/ma048258n

[B44] Peter K. T. Johns A. J. Myung N. V. Cwiertny D. M. (2017). Functionalized polymer-iron oxide hybrid nanofibers: electrospun filtration devices for metal oxyanion removal. Water Res. 117, 207–217. 10.1016/j.watres.2017.04.007 28399482

[B45] Porubská M. Szöllős O. Kóňová A. Janigová I. Jašková M. Jomová K. (2012). FTIR spectroscopy study of polyamide-6 irradiated by electron and proton beams. Polym. Degrad. Stab. 97 (4), 523–531. 10.1016/j.polymdegradstab.2012.01.017

[B46] Qiu H. Liang C. Yu J. Zhang Q. Song M. Chen F. (2017). Preferable phosphate sequestration by nano-La(III) (Hydr)Oxides modified wheat straw with excellent properties in regeneration. Chem. Eng. J. 315, 345–354. 10.1016/j.cej.2017.01.043

[B47] Radusch H.-J. Stolp M. Androsch R. (1994). Structure and temperature-induced structural changes of various polyamides. Polymer 35 (16), 3568–3571. 10.1016/0032-3861(94)90926-1

[B48] Rajak D. K. Pagar D. D. Menezes P. L. Linul E. (2019). Fiber-reinforced polymer composites: manufacturing, properties, and applications. Polymers 11 (10), 1667. 10.3390/polym11101667 31614875 PMC6835861

[B49] Rajak D. K. Wagh P. H. Linul E. (2022). A review on synthetic fibers for polymer matrix composites: performance, failure modes and applications. Materials 15 (14), 4790. 10.3390/ma15144790 35888257 PMC9321205

[B50] Ramaswamy S. Clarke L. I. Gorga R. E. (2011). Morphological, mechanical, and electrical properties as a function of thermal bonding in electrospun nanocomposites. Polymer 52 (14), 3183–3189. 10.1016/j.polymer.2011.05.023

[B51] Ramesh C. (1999). New crystalline transitions in nylons 4,6, 6,10, and 6,12 using high temperature X-ray diffraction studies. Macromolecules 32 (11), 3721–3726. 10.1021/ma981284z

[B52] Ramesh C. Keller A. Eltink S. J. E. A. (1994). Studies on the crystallization and melting of nylon-6,6: 1. The dependence of the brill transition on the crystallization temperature. Polymer 35 (12), 2483–2487. 10.1016/0032-3861(94)90367-0

[B53] Rayjadhav S. B. Kubade P. R. (2024). “Polyamide: comprehensive insights into types, chemical foundations, blending techniques and versatile applications,” in High-performance sustainable materials and structures. Editors Lazar P. Palani I. A. Kumar M. (Cham: Springer Nature Switzerland), 407–425. 10.1007/978-3-031-72527-2_30

[B54] Roslan N. S. A. Abdul Hamid N. Md Isa M. H. Muhammad N. Mansor M. R. Abdul Munajat N. (2018). Nylon electrospun nanofibre water filtration media for wastewater treatment. Mater. Res. Express 5 (10), 105010. 10.1088/2053-1591/aada94

[B55] Sanchaniya J. V. Lasenko I. Kanukuntala S. P. Smogor H. Viluma-Gudmona A. Krasnikovs A. (2023). Mechanical and thermal characterization of annealed oriented PAN nanofibers. Polym. (Basel) 15 (15), 3287. 10.3390/polym15153287 PMC10422648 37571181

[B56] Sheng J. Li Y. Wang X. Si Y. Yu J. Ding B. (2016). Thermal inter-fiber adhesion of the polyacrylonitrile/fluorinated polyurethane nanofibrous membranes with enhanced waterproof-breathable performance. Sep. Purif. Technol. 158, 53–61. 10.1016/j.seppur.2015.11.046

[B57] Skinner M. (2022). Wetland phosphorus dynamics and phosphorus removal potential. Water Environ. Res. 94 (10), e10799. 10.1002/wer.10799 36259138

[B58] Smith V. H. Tilman G. D. Nekola J. C. E. (1999). Eutrophication: impacts of excess nutrient inputs on freshwater, marine, and terrestrial ecosystems. Environ. Pollut. 100 (1–3), 179–196. 10.1016/s0269-7491(99)00091-3 15093117

[B60] Sridhara P. K. Masso F. Olsén P. Vilaseca F. (2021). Strong polyamide-6 nanocomposites with cellulose nanofibers mediated by green solvent mixtures. Nanomaterials 11 (8), 2127. 10.3390/nano11082127 34443955 PMC8401965

[B61] Srithep Y. Nealey P. Turng L.-S. (2013). Effects of annealing time and temperature on the crystallinity and heat resistance behavior of injection-molded poly(lactic acid). Polym. Eng. and Sci. 53 (3), 580–588. 10.1002/pen.23304

[B62] Su K.-H. Lin J.-H. Lin C.-C. (2007). Influence of reprocessing on the mechanical properties and structure of polyamide 6. J. Mater. Process. Technol. 192–193, 532–538. 10.1016/j.jmatprotec.2007.04.056

[B63] Sun J. Gao A. Wang X. Xu X. Song J. (2020). Removal of phosphorus from wastewater by different morphological alumina. Molecules 25 (13), 3092. 10.3390/molecules25133092 32645944 PMC7412428

[B64] Vasanthan N. (2009). “Polyamide fiber formation: structure, properties and characterization,” in Handbook of textile fibre structure. Editors Eichhorn S. J. Hearle J. W. S. Jaffe M. Kikutani T. (Woodhead Publishing Series in Textiles; Swaston, Cambridge, England: Woodhead Publishing), 1, 232–256. 10.1533/9781845696504.2.232

[B65] Vasanthan N. Salem D. R. (2001). FTIR spectroscopic characterization of structural changes in polyamide-6 fibers during annealing and drawing. J. Polym. Sci. Part B Polym. Phys. 39 (5), 536–547. 10.1002/1099-0488(20010301)39:5<536::AID-POLB1027>3.0.CO;2-8

[B66] Wang C. Yu S. Cwiertny D. M. Yin Y. Myung N. V. (2021). Phosphate removal using surface enriched hematite and tetra-n-butylammonium bromide incorporated polyacrylonitrile composite nanofibers. Sci. Total Environ. 770, 145364. 10.1016/j.scitotenv.2021.145364 33736373

[B67] Wang X. Hsiao B. S. (2016). Electrospun nanofiber membranes. Curr. Opin. Chem. Eng. 12, 62–81. 10.1016/j.coche.2016.03.001

[B68] Wang Z. Cai N. Dai Q. Li C. Hou D. Luo X. (2014). Effect of thermal annealing on mechanical properties of polyelectrolyte complex nanofiber membranes. Fibers Polym. 15 (7), 1406–1413. 10.1007/s12221-014-1406-2

[B69] Zarrini K. Rahimi A. A. Alihosseini F. Fashandi H. (2017). Highly efficient dye adsorbent based on polyaniline-coated nylon-6 nanofibers. J. Clean. Prod. 142, 3645–3654. 10.1016/j.jclepro.2016.10.103

[B70] Zhang Y. Zhang Y. Liu S. Huang A. Chi Z. Xu J. (2011). Phase stability and melting behavior of the α and γ phases of Nylon 6. J. Appl. Polym. Sci. 120 (4), 1885–1891. 10.1002/app.33047

[B71] Zhu F. Zheng Y.-M. Zhang B.-G. Dai Y.-R. (2021). A critical review on the electrospun nanofibrous membranes for the adsorption of heavy metals in water treatment. J. Hazard. Mater. 401, 123608. 10.1016/j.jhazmat.2020.123608 33113718

