# Supplementary material for: Thermally treated lanthanum oxide nanoparticles-embedded polyamide composite nanofiber membrane for enhanced mechanical properties and phosphorus adsorption kinetics
Source: Front Chem. 2025 Jul 24;13:1630889. doi: 10.3389/fchem.2025.1630889 (PMC12328448; doi:10.3389/fchem.2025.1630889)
Supplement: Supplementary file 1 [file DataSheet1.docx]

**Supplementary Material**

**Thermally Treated Lanthanum Oxide Nanoparticles-Embedded Polyamide Composite Nanofiber Membrane for Enhanced Mechanical Properties and Phosphorus Adsorption Kinetics**

Yun Young Choi^1^ David M. Cwiertny^2^ and Nosang V. Myung^1,3*^

^1^Department of Chemical and Biomolecular Engineering,

University of Notre Dame, Notre Dame, IN 46556, United States

^2^Department of Civil and Environmental Engineering,

University of Iowa, 4105 Seamans Center, Iowa City, IA 52242, USA.

^3^Department of Chemistry and Biochemistry

University of Notre Dame, Notre Dame, IN 46556, United States

Table S1. Solution properties of PA6/La_2_O_3_/TBAB NF with various solvents.

| Sample # | Solution Properties | | | | Nanofiber Properties | | | |
| --- | --- | --- | --- | --- | --- | --- | --- | --- |
|  | Solvents | Viscosity  [cP] | Surface Tension [dynes/cm] | Electrical Conductivity [μS/cm] | Average fiber diameter  [nm] | | Fiber fraction [μm^2^/μm^2^] | Bead density [beads/μm^2^] |
| 1 | Formic Acid | 1664 | 37.9 | 4770 | 65 ± 19 | 0.84 | | 0.16 |
| 2 | Acetone: TFA  (60:40 mol%) | 90.12 | 21.4 | 1838 | 97 ± 31 | 0.93 | | 0.07 |
| 3 | HFIP | 69.19 | 20.3 | 235.9 | 162 ± 36 | 0.98 | | 0.02 |


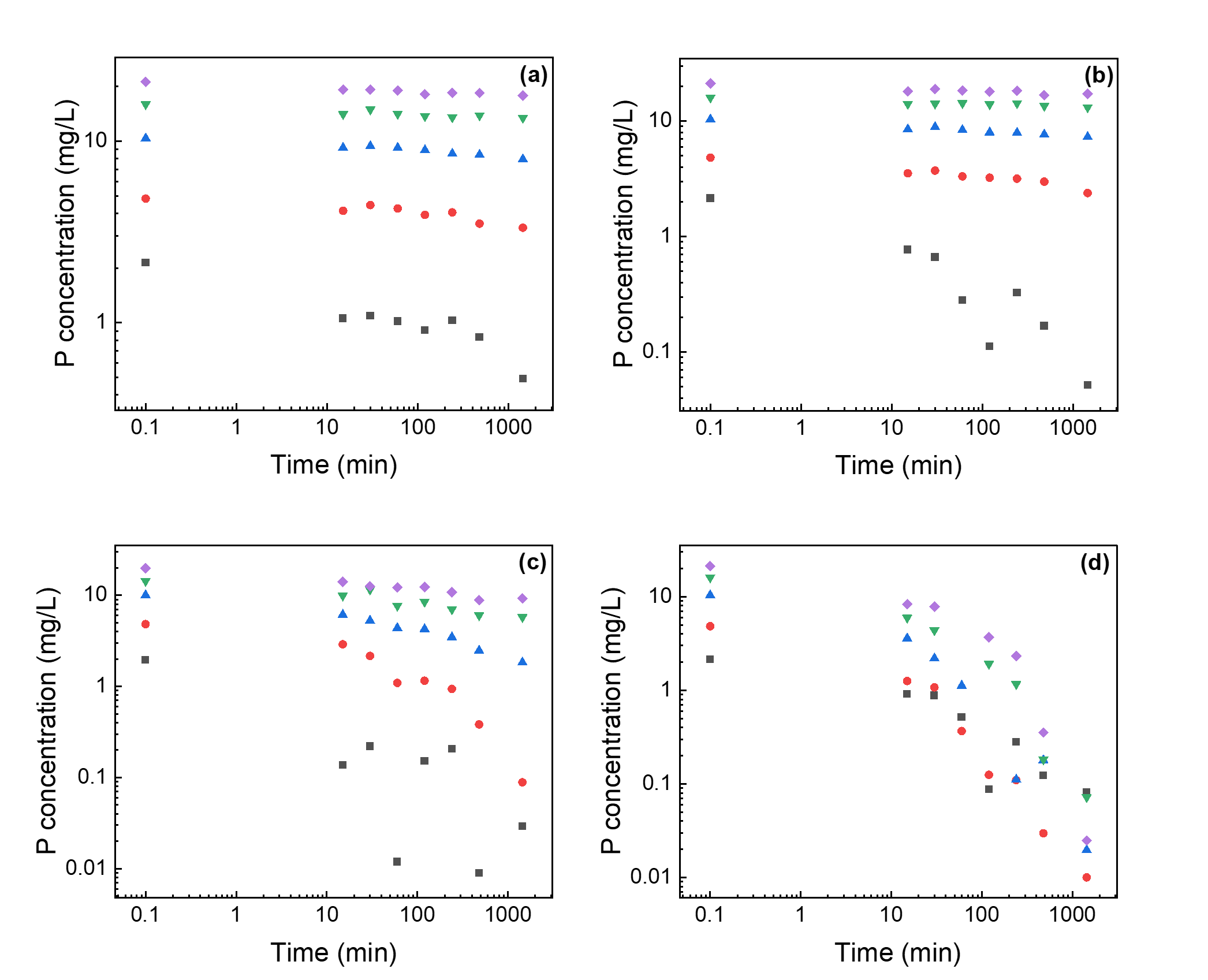


Figure S2. Phosphate removal using various metal oxide nanoparticles: (a) alumina, (b) cerium oxide, (c) iron oxides, and (d) lanthanum oxide at various initial concentrations. Adsorbent dosage: 1g/L.


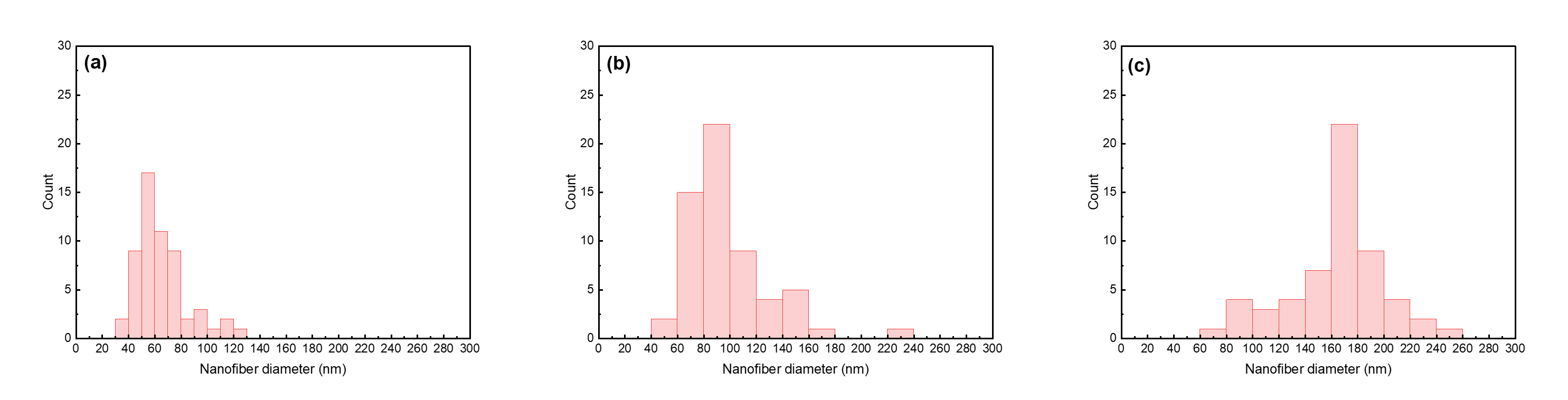


Figure S1. Size distribution of PA6/La_2_O_3_/TBAB NF membrane with various solvents:

(a) formic acid, (b) Ace: TFA (60:40 mol%), and (c) HFIP.


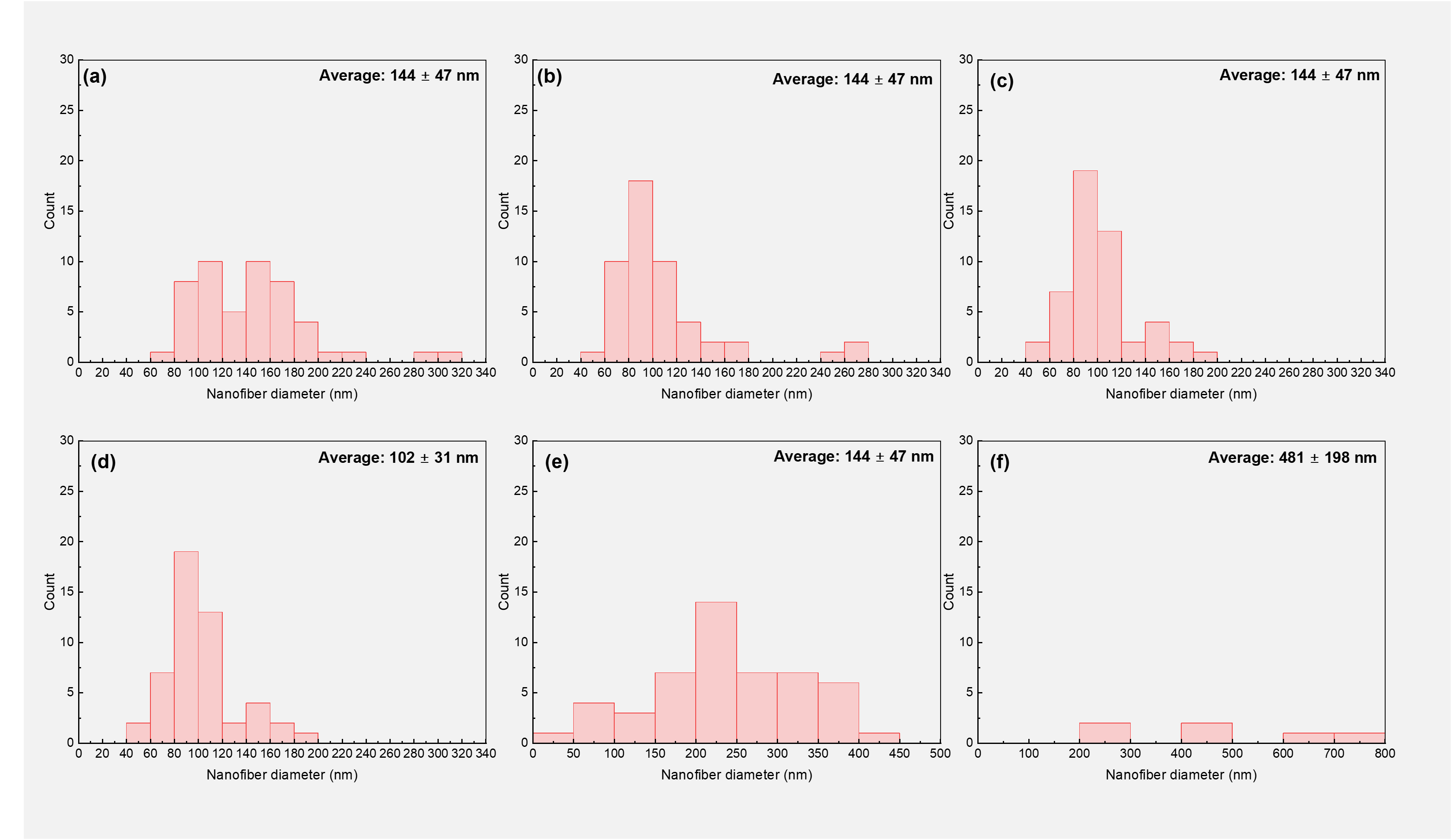


Figure S4. Size distribution of PA6/La_2_O_3_/TBAB NF membrane (a) as spun, and annealed at various temperatures: (b) 80^°^C, (c) 100^°^C, (d) 120^°^C, (e) 140^°^C, and (f) 200^°^C.


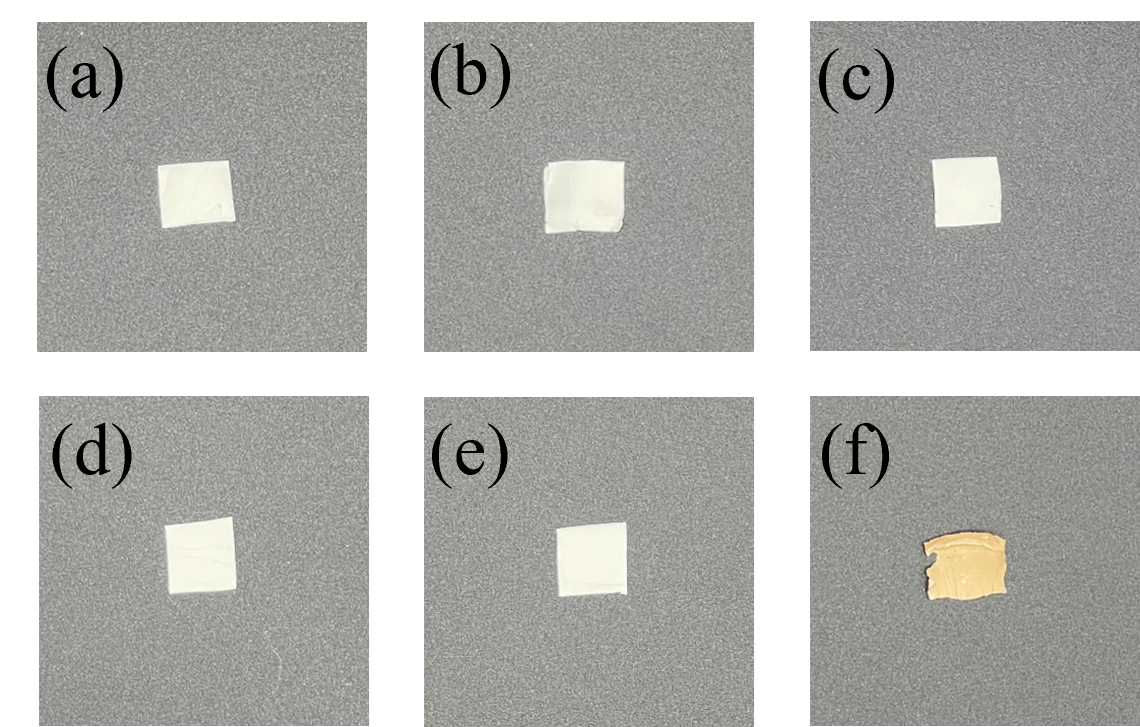


Figure S3. Optical images of PA6/La_2_O_3_/TBAB NF membrane (a) as spun, and annealed at various temperatures: (b) 80^°^C, (c) 100^°^C, (d) 120^°^C, (e) 140^°^C, and (f) 200^°^C.
